# Recognition of sites of functional specialisation in all known eukaryotic protein kinase families

**DOI:** 10.1371/journal.pcbi.1005975

**Published:** 2018-02-13

**Authors:** Raju Kalaivani, Raju Reema, Narayanaswamy Srinivasan

**Affiliations:** Molecular Biophysics Unit, Indian Institute of Science, Bangalore, Karnataka, India; University College London, UNITED KINGDOM

## Abstract

The conserved function of protein phosphorylation, catalysed by members of protein kinase superfamily, is regulated in different ways in different kinase families. Further, differences in activating triggers, cellular localisation, domain architecture and substrate specificity between kinase families are also well known. While the transfer of γ-phosphate from ATP to the hydroxyl group of Ser/Thr/Tyr is mediated by a conserved Asp, the characteristic functional and regulatory sites are specialized at the level of families or sub-families. Such family-specific sites of functional specialization are unknown for most families of kinases. In this work, we systematically identify the family-specific residue features by comparing the extent of conservation of physicochemical properties, Shannon entropy and statistical probability of residue distributions between families of kinases. An integrated discriminatory score, which combines these three features, is developed to demarcate the functionally specialized sites in a kinase family from other sites. We achieved an area under ROC curve of 0.992 for the discrimination of kinase families. Our approach was extensively tested on well-studied families CDK and MAPK, wherein specific protein interaction sites and substrate recognition sites were successfully detected (*p-value* < 0.05). We also find that the known family-specific oncogenic driver mutation sites were scored high by our method. The method was applied to all known kinases encompassing 107 families from diverse eukaryotic organisms leading to a comprehensive list of family-specific functional sites. Apart from other uses, our method facilitates identification of specific protein interaction sites and drug target sites in a kinase family.

## Author summary

Protein kinases are molecular switches that destine crucial decision points in cell signalling pathways. Consequently, they are implicated in the normal functioning of a cell as well as in various cancers if mutated. Kinases constitute a large and diverse superfamily with conserved 3-dimensional structure and catalytic function. Despite the monotony, individual kinase families differ extensively in their cognate substrates, binding partners and mode of regulation. The determinants of these specific characteristics are unknown for most kinase families. Using an integrated computational method, tested successfully on known cases, we propose a comprehensive list of functionally-specialized sites in all known kinase families. Such knowledge allows for understanding of mechanistic basis of regulation and tinkering of functions specific to a kinase family.

## Introduction

Protein kinases, as key regulators of cellular functions, are among the largest and most diverse protein superfamilies known [1,2]. On account of their phosphotransfer function to a Ser / Thr / Tyr residue in eukaryotes, they are also known as STY kinases. Congruent to their function as molecular switches that determine outcome at critical decision points of cell signalling pathways, their indispensable nature is reflected by the conservation of at least 51 unique kinase families across phyla, from yeast to mammals [3]. During the course of divergent evolution, the broad catalytic function and the 3-dimensional fold are well conserved [4]. Despite the monotony, kinases exhibit high diversity in terms of differences in activating triggers [5–8], regulatory mechanisms [9,10], cellular localisation [11–13], domain architectures [1] and substrate specificity [14]. In this context of dualism of similarity and differences, the modules responsible for common and preserved features, like, ATP binding [15], phosphotransfer [16] and 3-dimensional conformation of active state [17] are well known. However, the correlates of kinase-specific functional and regulatory attributes, which differentiate one kinase from another, are not completely understood. Indeed, for many kinases the sites of functional specialization is yet unknown.

In the present study, we aim to identify the sites of functional specialisation in all known eukaryotic protein kinase families. These are residues involved in specific protein-protein interactions, cognate substrate recognition, response to specific signals, etc., and thus are the defining and discriminating attributes of the corresponding kinase. Clearly, knowledge of such sites finds immense application in designing kinase-specific inhibitors, protein engineering and recognition of interaction partners. Such sites should ideally be identified by traditional experimental methods like mutation studies and structural analyses using X-ray diffraction; but these are evidently slow processes as in-depth information on family-specific functional sites is so far known only for a few kinase families such as PKA [18], Src [19], MAPK [20] and CDK [21,22]. In this scenario, we have analysed the sequences of all known STY kinases, comprehensively studied the conservation patterns within and across kinase families, devised a unified scheme and identified kinase family-specific functional sites in each of them.

Residues of functional specialisation in a particular kinase family, say PKA, are by definition, crucial for PKA-specific functions and regulatory mechanisms, and thus are expected to be conserved in all PKAs [23]. Additionally, since the associated function / regulation itself is PKA-specific, evolutionary pressure for conservation of these sites exists selectively in PKA kinases. As a result, such sites also possess the discriminatory ability to distinguish PKA from other kinase families like PKC and Src. Following this rationale, we use two cardinal properties of family-specific functional sites, *viz*., (i) differential conservation and (ii) discriminatory ability, to identify them.

In the past, several studies have attempted to delineate functionally characterised residues in a family of homologous proteins [24–26]. Some methods like evolutionary trace analysis [27] and energetics-based predictions [28] rely on protein structure to identify protein-ligand and protein-protein interfaces. Other methods perform hierarchical analysis [29,30], statistical analysis [31–34], ortholog and paralog investigations [35,36], calculation of rate of evolution [37] and log-likelihood analyses [38] of protein sequences to identify specificity determinants [39]. These studies may measure absolute conservation of amino acids [27,40], conservation of physicochemical properties [29], correlated mutations [41,42], Shannon entropy and mutual information [35,36,43–50], and probability [51,52], among others [30,46,53–58]. However, these methods followed an all-or-none approach, in which a residue is labelled either functional or unimportant. The emerging picture of modularity, within and outside of a protein domain, is increasingly pointing towards a continuum of functional importance of residues and regulatory features [18]. Further, these studies carry the limitations of the individual quantification methods used. Most protein kinase studies have considered the entire superfamily of protein kinases as one cluster [59], while others looked into specificity determining residues at the group level [60]. In the current study, we propose an integrated scheme which uses the advantages of several methods (conservation of physicochemical property, Shannon entropy and random probability distribution) and scores the sites on a continuous scale of their functional / regulatory specificity at the family level.

We systematically compiled a dataset of 5488 kinase catalytic domain sequences belonging to 107 distinct kinase ‘families’ [61,62]. After aligning them into a single multiple sequence alignment, we comparatively analysed the amino acid distributions in topologically equivalent positions of different families. Based on 3 different analytical measures, we identified family-specific functional sites that are differentially conserved in each of the 107 families. By maximising the discriminability between the kinase families, we integrated the results of the three measures and devised a unified scoring scheme called ID_score. We assessed the competence of this method by testing its ability to (i) cluster kinase sequences into groups and families, (ii) aid a linear classifier in predicting the family of the kinase, and (iii) identify experimentally determined kinase-specific functional sites like protein-protein interaction sites in CDK, substrate recognition sites in MAPK and specific cancer-causing driver mutation sites. Finally, we recognise the sites of functional specialisation in all known kinase families and demonstrate one of the applications of this method in the prediction of specific protein-protein interaction sites. In summary, we developed an integrated discriminatory method to identify regions of functional specialisation, validated the results for known cases and applied the method to all known kinase families to present an exhaustive list of sites of functional specialization in all the kinases involved in this study.

## Results

The results of the study are organised into four major sections: (i) dataset curation, explaining the method of selection and organisation of STY kinase catalytic domain sequences, (ii) method development, detailing the rationale and protocol to identify differentially conserved sites in kinases and maximise the discriminability among them, (iii) method assessment and validation, elucidating its performance by application to known sites of functional specialisation in a few well studied kinases, and (iv) application, demonstrating a feasible avenue for practical use of the method and applying it to all known kinases.

### Dataset curation

**Fig 1:**
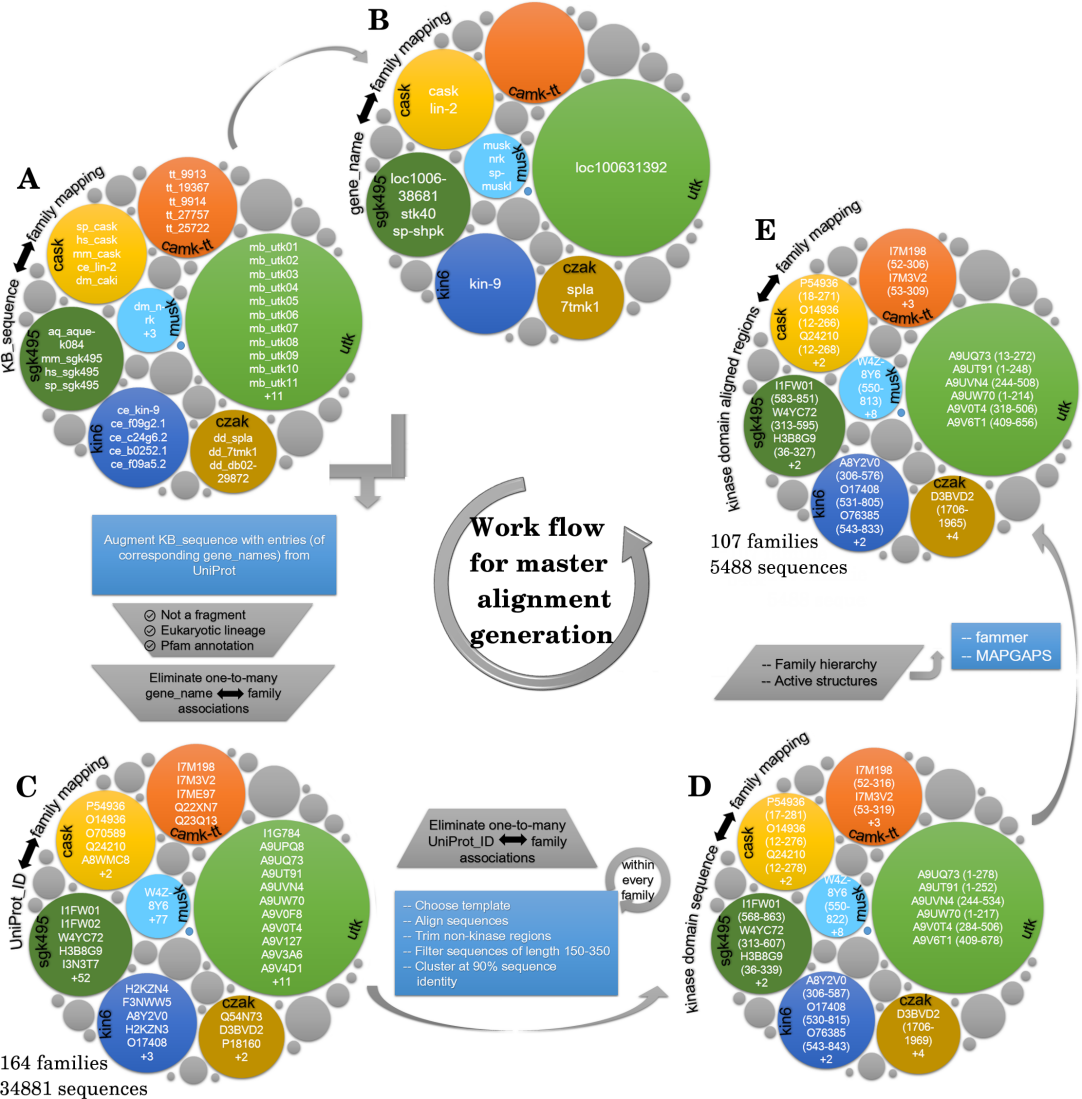
Curation of STY kinase catalytic domain sequence dataset. Depicted is a schematic of the work flow followed to achieve a master alignment of all known kinase catalytic domains. The sequence-to-family association for kinases was first retrieved from KinBase (KB), and illustrated in (A). The circles represent different KB families and the entries within correspond to sequences, shown by KB identifiers, belonging to the family. This is exemplified by 7 representative kinase families cask (*yellow*), camk-tt (*orange*), musk (*light blue*), utk (*light green*), sgk495 (*dark green*), kin6 (*dark blue*) and czak (*brown*). The genes of the kinases classified by KB were retrieved to form a gene-to-family association (B). Protein products of the identified genes were then collated from all eukaryotes and augmented to the existing KB_sequence-to-family mapping to achieve an extended classification of all kinases in UniProt into families (C). Care was taken to include only non-fragments and sequences with kinase domain annotation. Kinase catalytic domain regions were then extracted from the full length protein to form the kinase domain sequence-to-family association (D), such that no two sequences within a family share a sequence identity of >90%. The UniProt IDs, along with the kinase domain extents, are enlisted within each family. All these sequences, across families, were aligned into a single multiple sequence alignment (See Alignment in Methods), which served as an input to the ID_score method. The final dataset of 5488 kinases of 107 families in the multiple sequence alignment are represented by their UniProt IDs and kinase domain extents in (E).

The primary rationale behind our method is to recognize sites in the kinase catalytic domain that are conserved uniquely within a family of kinases [27]. This passively assumes the existence of a reliable system of classification of all known STY kinases into families. Such an empirical and curated system of kinase classification developed after a series of comprehensive studies [3,63–66], KinBase (KB), is illustrated in Fig 1A. This system of hierarchical classification clusters the STY kinases broadly into ‘groups’ and more finely into ‘families’. For demonstration purpose, 7 randomly selected kinase families (cask, camk-tt, musk, utk, sgk495, kin6 and czak) are depicted as coloured circles (*yellow*, *orange*, *light blue*, *light green*, *dark green*, *dark blue* and *brown* respectively) among other families (*gray circles*) (Fig 1A). Kinases belonging to these families, as identified by KB, are enlisted inside the corresponding circles.

We note that the KB system of classification, and thus the KB_sequence-to-family mapping (Fig 1A), is available for kinases from as many as 15 species. However, in the exigency of the study, it is vital to construct a dataset with kinase sequences as diverse as possible within each family so as to distinguish sites that are truly conserved through the course of evolution from those that accommodate variation without affecting the stability and function of the protein. Thus, we aim to augment the KB_sequence-to-family mapping (Fig 1A) with additional sequences from other organisms/phyla. To this end, we first identified the genes of every sequence in the existing mapping by individual BLASTs [67] against UniProt [68], and curated a gene-to-family mapping (Fig 1B). Gene names of kinases belonging to different families are enlisted inside the corresponding family circles in Fig 1B. We note that gene names could not be identified for a few uncharacterised sequences (e.g., sequences from family camk-tt) in the KB mapping. We then enriched the KB_sequence-to-family mapping with sequences of corresponding genes from other phyla / organisms (Fig 1C). While doing so, care was taken to include only non-fragment sequences of eukaryotic lineage with kinase domain annotation (Pfam IDs: PF00069 and/or PF007714) [69]. In the cases of ambiguous association of the same gene to multiple families in KinBase, which is rare, kinase sequences of the corresponding gene from all organisms were eliminated from the dataset. This resulted in UniProt_ID-to-family mapping of 34,881 kinase sequences into 164 families (Fig 1C), consisting of sequences originally present in KB as well as their orthologues in other species (full dataset available as S1 File). In Fig 1C, the UniProt IDs of kinase sequences belonging to different families are enlisted in the corresponding family circles.

The dataset was further subjected to filters and constraints (see Dataset sub-section in Methods section) to eliminate ambiguity and extract the kinase catalytic domain region from full length sequence, as described below. After eliminating sequences with ambiguous associations with multiple families, a template sequence was chosen for every family. Choice of the template sequence was based on (i) availability of information on the boundary (region) of kinase catalytic domain in the full length sequence [70], and (ii) structurally well-studied nature of the sequence as reflected by the highest number of available crystal structures for the kinase when compared to other kinases within the family. In case of absence of crystal structures for a kinase family, a sequence with known boundary of kinase catalytic domain was randomly chosen as the template. Next, all sequences in a family were aligned using MAFFT [71] and the kinase catalytic domains were extracted for all sequences based on the boundary of the catalytic domain of the template sequence. Kinase catalytic domain sequences thus extracted were clustered at 90% sequence identity [72] to remove any bias or redundancy in the dataset. In Fig 1D, the unbiased dataset of kinase domain sequence-to-family mapping is illustrated, wherein the UniProt IDs and the boundaries of kinase domain are enlisted in the corresponding family circles.

Finally, all the kinase domain sequences across families were aligned into a single multiple sequence alignment (See Alignment section in Methods) of 5488 sequences from 107 families of 7 distinct groups, which serves as an input to our method. During the alignment process, a few sequences that could not be aligned confidently were discarded and the kinase catalytic domain boundary was further pruned in order to trim the flanking gap regions in the termini. In Fig 1E, the UniProt IDs and the boundaries of kinase domains, as present in the final multiple sequence alignment, are enlisted in the corresponding family circles (full alignment available as S2 File).

### Development of a method to predict sites of functional specialization

By parsing the alignment generated in the previous step, we set out to pinpoint the uniquely conserved sites that maximise the discriminability of the family from the rest. This rationale calls for a systematic position-wise comparison of the residues populating the family of interest with those in the other families in a quantitative manner. In the past, several attributes were shown to be useful measures to quantify the residue distributions [27,29,35,40,43,44,49,52,53]. We used 3 such attributes, conservation of physicochemical property (*pc*), Shannon’s entropy (*ent*) and statistical probability (*prob*), to measure the similarities and differences in residue distributions between families. Later, we integrated the results of the 3 measures and devised a single scheme (ID_score) that scores the kinase residues in a manner that is reflective of the uniqueness of the site to the family.

#### Pipeline of the method

**Fig 2:**
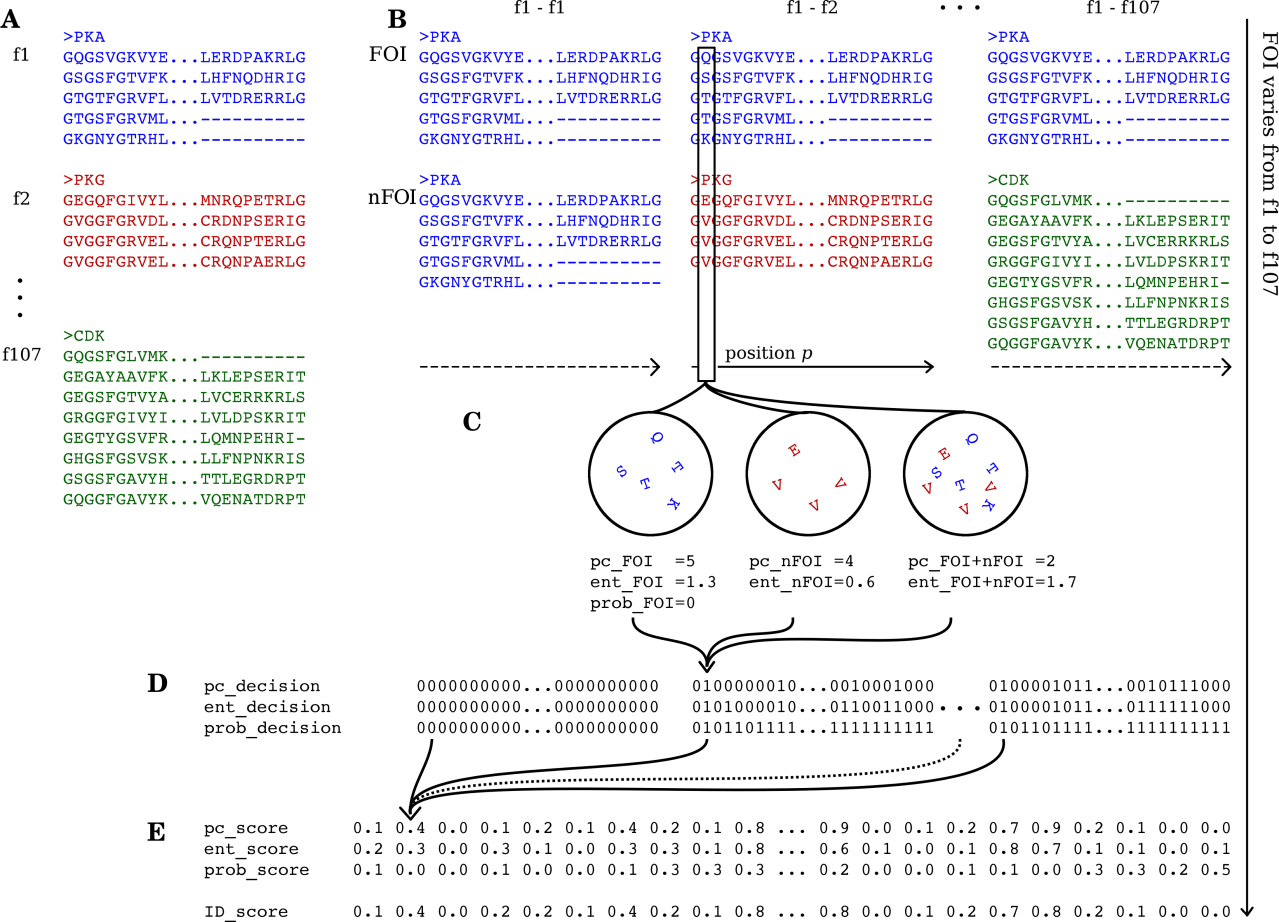
Pipeline of ID_score method. Shown is a schematic representation of the ID_score method to identify characteristic sites in kinase families. (A) A representative fragment of the master alignment is shown illustrating 3 of the 107 families, f1 (PKA, *blue*), f2 (PKG, *red*) and f107 (CDK, *green*). Considering f1 as the family of interest (FOI), for which characteristic sites are to be identified, pair wise comparisons of the FOI f1 with every family in the dataset (nFOIs), viz., f1-f1, f1-f2, …, f1-f107, are carried out (B). During this procedure, every alignment position in FOI f1 is compared with the corresponding position in each of the 107 families. An example alignment position *p* ((B), *boxed*) is depicted, showing the amino acids populating the categories FOI, nFOI and FOI+nFOI (C). Three measures, conservation of physicochemical property (*pc*), Shannon’s entropy (*ent*) and statistical probability (*prob*) are quantified for the different categories, based on which every position *p* in FOI is declared unique (1) or otherwise (0) with respect to the corresponding position in every nFOI. These form the *pc_decision*, *ent_decision* and *prob_decision* respectively (D), which when averaged across the 107 pair wise comparisons gives rise to *pc_score*, *ent_score* and *prob_score* (E). A linear weighted combination of the individual measures *pc_score*, *ent_score* and *prob_score* is achieved by optimisation (See Results) to obtain ID_score, which scores every alignment position in f1 on a scale of 0 to 1, with 0 representing no specificity and 1 representing high specificity. This protocol is repeated, considering every family in the dataset as the FOI to identify characteristic sites in them.

**Fig 3:**
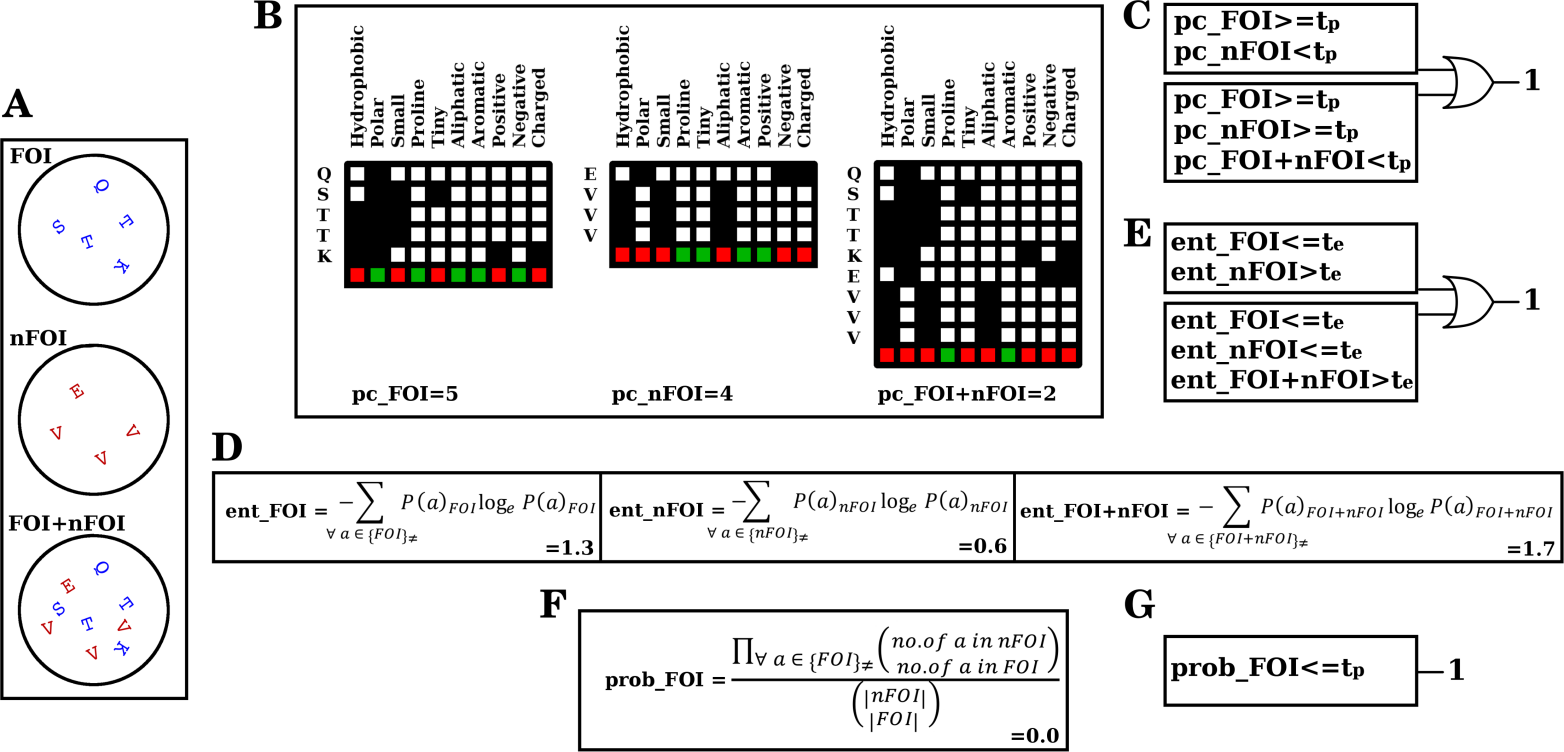
Three measures to decide the uniqueness of a position *p*. The amino acids populating an example position *p* in categories FOI, nFOI and FOI+nFOI, as seen in Fig 2, is shown (A). Every amino acid in each of the categories is assessed for the presence (B, *filled black box*) or absence (B, *unfilled white box*) of 10 physicochemical properties. The properties that are uniformly present or absent (*green*) in every amino acid in FOI, nFOI and FOI_nFOI are counted and called *pc_FOI*, *pc_nFOI* and *pc_FOI+nFOI* respectively (B). In case of higher conservation of physicochemical properties in FOI or differential nature of physicochemical properties conserved in FOI, a decision of uniqueness (*pc_decision*) is declared to be 1 (C). Shannon’s entropy for the distribution of amino acids in FOI, nFOI and FOI+nFOI are determined and called *ent_FOI*, *ent_nFOI* and *ent_FOI+nFOI* respectively (D). (E) In case of lower randomness in FOI or differential nature of low randomness in FOI, the position is declared unique (*ent_decision* = 1). The probability of drawing the distribution of amino acids in FOI upon repeated draws from the amino acid sample in nFOI, without replacement, is calculated and called *prob_FOI* (F). (G) In case the calculated probability is sufficiently low, the position is considered unique (*prob_decision* = 1). t_p_, t_e_ and t_s_ are the thresholds operative for the three measures *pc*, *ent* and *prob* respectively (See text for details).

The master alignment of 5488 sequences belonging to 107 families, indexed from f1 to f107, is schematised in Fig 2A depicting parts of sequences of families f1 (PKA, *blue*), f2 (PKG, *red*), …, f107 (CDK, *green*). It should be noted that the alignment depicted in Fig 2A is only illustrative, and does not reflect the entire master alignment. Also, for purpose of clarity, we outline the pipeline of the method that identifies family-specific sites for a family of interest (FOI) f1. Although the description in the section and illustration in Figs 2 and 3 are limited to f1, the entire procedure was repeated considering every family (f1, f2, …, f107) as the FOI in order to identify family-specific sites in each of them.

An overt means to identify family-specific sites would be to compare the residues at every alignment position *p* in the FOI f1 with those at the same position in all the other families. However, f1 may be evolutionarily and functionally more closely related to a few families (say, f2 and f3) than to others (say, f4, f5, …, f107). Thus, some attributes of *p* would be commonly shared with that of the closely related families alone. As a result, upon comparison of *p* in the FOI f1 with that of all the other families put together, f2-f107, quantification of the uniquely conserved nature of *p* is not straightforward. We resolved this by performing pair wise comparisons of the FOI with every family in the dataset (nFOIs), *viz*., comparisons f1-f1, f1-f2, …, f1-f107, as illustrated in Fig 2B. The justification is that a truly family-specific position in f1 will be differentially conserved in f1 in most / all of the pair wise comparisons. On the other hand, a position in f1, say, from the ATP binding loop, whose function is globally conserved across all kinase families, will be differentially conserved only in a few / none of the pair wise comparisons. Thus, the fraction of pair wise comparisons in which *p* is differentially conserved give a direct weight, on a scale of 0 to 1, indicating the magnitude of uniqueness of the position to the FOI. Thus, every alignment position of the FOI was pair wise-compared with the corresponding position of each of the 107 families in the dataset (nFOIs) as shown in Fig 2B.

Comparison of a position *p* in FOI f1 with that of, say, an nFOI f2 (Fig 2B, *boxed position*) was made by analysing the residues that populate the family of interest (FOI), the family being compared with (nFOI) and both the families considered together (FOI+nFOI), as illustrated in Fig 2C. It should be noted that, in order to correct for any inaccuracy in the alignment, the residues in position *p* in a family at a frequency less than 8.5% were disregarded before subsequent analyses. The conservation of physicochemical properties in the 3 categories, FOI, nFOI and FOI+nFOI, is quantified (See Physicochemical measure section) and is denoted as *pc_FOI*, *pc_nFOI* and *pc_FOI+nFOI* respectively. These were then consolidated and a binary decision, as to whether (1) or not (0) the position *p* is differentially conserved in the FOI f1 when compared to the nFOI f2, with respect to conservation of physicochemical properties of the residues, was taken. Since the FOI f1 was pair wise compared with each of the 107 families in the dataset, we achieved 107 binary decisions (1 or 0) for an alignment position *p*, one for each pair wise comparison. This procedure was repeated considering every position in the master alignment as *p*, giving rise to *pc_decision* (Fig 2D). Likewise, *ent_decision* and *prob_decision* were also calculated by measuring Shannon entropy and random probability attributes of the residue distributions respectively (See Entropy measure and Probability measure sections). The 3 methods (*pc*, *ent* and *prob*) weigh the FOI against nFOIs using different sets of rules, and thus might result in different binary decisions for the same position.

We note that *pc_decision*, like *ent_decision* and *prob_decision*, of FOI f1 is simply a matrix of 1s and 0s of size 107xN, where N is the total number of alignment positions in the master alignment. This matrix contains a binary value for each pair wise comparison at every alignment position, indicating whether (1) or not (0) the particular position is differentially conserved when compared with a particular family. The binary values across the 107 pair wise comparisons were then averaged for every alignment position in f1 to get a *pc_score* (Fig 2E). In other words, *pc_score* of FOI f1 is a vector of length N, with values ranging between 0 and 1, with 0 indicating differential conservation in the position in none of the pair wise comparisons and 1 indicating differential conservation in all the pair wise comparisons. Likewise, *ent_score* and *prob_score* for FOI f1 were calculated, based on *ent_decision* and *prob_decision* respectively. Thus, we scored every alignment position in f1 on a scale of 0 to 1 using 3 different measures (Fig 2E). The 3 individual scores (*pc*, *ent* and *prob*) at every alignment position was then linearly combined (See Integrated discriminatory score section) to achieve an integrated score, or ID_score. ID_score for family f1 is a vector of length N, containing scores ranging between 0 and 1 for each alignment position, with 0 indicating no family-specificity and 1 indicating high family-specificity (data available as S3 File). The pipeline described above was repeated, considering each of the 107 families as FOI to identify family-specific sites in them.

#### Physicochemical measure (*pc_decision*)

This section explains how the conservation of physicochemical properties at a given alignment position *p* was quantified. Ten physicochemical properties were considered [29,73,74]: hydrophobic (I,L,V,C,A,G,M,F,Y,W,H,K,T), polar (C,Y,W,H,K,R,E,Q,D,N,S,T), small (V,C,A,G,D,N,S,T,P), Proline (P), tiny (C,A,G,S), aliphatic (I,L,V), aromatic (F,Y,W,H), positive (H,K,R), negative (E,D) and charged (H,K,R,E,D). Gaps (‘-‘) in the alignment and unknown amino acids (X) were regarded as having none of the 10 properties. The residues in FOI (Fig 3A) were scrutinised for the presence (Fig 3B, *1*^*st*^ *panel*, *filled black box*) or absence (Fig 3B, *1*^*st*^ *panel*, *unfilled white box*) of each of the 10 physicochemical properties. The number of physicochemical properties that are absolutely conserved in all the residues in FOI, nFOI and FOI+nFOI was counted and referred as *pc_FOI*, *pc_nFOI* and *pc_FOI+nFOI* respectively (Fig 3B, *green box*). It is to be noted that if a specific property is either uniformly present or uniformly absent in all the residues in a position, it is counted as a conserved property in the spirit that the presence or absence of that property is evolutionarily selected. The three category measures *pc_FOI*, *pc_nFOI* and *pc_FOI+nFOI*, representing the number of absolutely conserved physicochemical properties in a given position *p* in FOI, nFOI and FOI+nFOI respectively, could each take integral values between 0 and 10.

Based on the physicochemical measure, there are two scenarios in which position *p* could be declared differentially conserved in FOI when compared to the nFOI (Fig 3C): (i) when there is conservation of physicochemical properties in FOI but not in nFOI, i.e., if *pc_FOI* is greater than or equal to a threshold value t_p_ (*pc_FOI* ≥ *t*_*p*_) and *pc_nFOI* < *t*_*p*_, or (ii) when both FOI and nFOI have conserved physicochemical properties, but the properties conserved in them are different, i.e., *pc_FOI* ≥ *t*_*p*_, *pc_nFOI* ≥ *t*_*p*_ and *pc_FOI* + *nFOI* < *t*_*p*_. Thus,

$$pc\_decision(nFOI,p)=\left\{ \begin{array}{ll} 1, & \;pc\_FOI\geq t_{p},\;pc\_nFOI<t_{p}\\ 1, & \;pc\_FOI\geq t_{p},\;pc\_nFOI\geq t_{p},\;pc\_FOI+nFOI<t_{p}\\ 0, & \;else \end{array}\right.$$

where *pc_decision*(*nFOI*,*p*) is the binary decision of whether the position *p* in FOI is differentially conserved in comparison with the corresponding position in an nFOI; and *t*_*p*_ is the threshold number of conserved physicochemical properties (See Determination of threshold values section).

#### Entropy measure (*ent_decision*)

This measure is used to quantify the difference in a given position *p* between the FOI and nFOI in terms of randomness of the residues populating with the position [43,75,76]. Shannon entropy was calculated for the FOI, nFOI and FOI+nFOI categories by measuring the frequency of occurrence of residues in FOI, nFOI and FOI+nFOI respectively (Fig 3D). These are accordingly called *ent_FOI*, *ent_nFOI*, and *ent_FOI+nFOI*. In this measure, the frequencies of each of the 22 possible values (20 amino acids, unknown (X) and gap (‘-‘)) were considered individually, irrespective of any physicochemical relatedness between them.

$$\begin{array}{l} ent\_FOI\;=-\sum_{\forall \;a\in {\left\{FOI\right\}}_{\neq }}\;{P(a)}_{FOI}\log_{e}{P(a)}_{FOI}\\ ent\_nFOI\;=-\sum_{\forall \;a\in {\left\{nFOI\right\}}_{\neq }}{\;P(a)}_{nFOI}\log_{e}{P(a)}_{nFOI}\\ ent\_FOI+nFOI\;=-\sum_{\forall \;a\in {\left\{FOI+nFOI\right\}}_{\neq }}{P(a)}_{FOI+nFOI}\log_{e}{P(a)}_{FOI+nFOI} \end{array}$$

where {*FOI*}_≠_, {*nFOI*}_≠_ and {*FOI* + *nFOI*}_≠_ are the sets of non-redundant amino acids in position *p* of FOI, nFOI and FOI+nFOI respectively; and *P*(*a*)_*FOI*_, *P*(*a*)_*nFOI*_ and *P*(*a*)_*FOI*+*nFOI*_ are the frequencies of occurrence of *a* in position *p* of FOI, nFOI and FOI+nFOI. The three category measures *ent_FOI*, *ent_nFOI* and *ent_FOI+nFOI*, representing the randomness associated with position *p* in FOI, nFOI and FOI+nFOI respectively, could each take values between 0 (absolute conservation of a single amino acid) and 3.09 (equal occurrence of all the 22 values).

Based on entropy measure, position *p* was declared differentially conserved in FOI when compared to the nFOI (Fig 3E): (i) if there was low randomness in FOI, but not in nFOI, i.e., ent_FOI is smaller than or equal to a threshold value t_e_ (*ent_FOI* ≤ *t*_*e*_)and *ent_nFOI* > *t*_*e*_, or (ii) if both FOI and nFOI had low randomness, but the residues conserved in them were different, i.e., *ent_FOI* ≤ *t*_*e*_, *ent_nFOI* ≤ *t*_*e*_ and *ent_FOI* + *nFOI* > *t*_*e*_.

$$ent\_decision(nFOI,p)=\left\{ \begin{array}{ll} 1, & \;ent\_FOI\leq t_{e},\;ent\_nFOI>t_{e}\\ \;1, & \;ent\_FOI\leq t_{e},\;ent\_nFOI\leq t_{e},\;ent\_FOI+nFOI>t_{e}\\ 0, & \;else \end{array}\right.$$

where *ent_decision*(*nFOI*, *p*) is the binary decision of whether the position *p* in FOI is differentially conserved in comparison with the corresponding position in an nFOI; and *t*_*e*_ is the threshold entropy (See Determination of threshold values section).

#### Probability measure (*prob_decision*)

This measure calculates the probability of obtaining the exact set of residues found in position *p* in FOI when one repeatedly draws, without replacement, from the set of residues in the same position of nFOI (Fig 3F). This score essentially captures the chance occurrence of residues in *p* of FOI from those at nFOI, and ranges between 0 and 1. Using classical statistics, we can calculate this probability, *prob_FOI*, as follows:

prob_FOI=∏∀a∈{FOI}≠(no.ofainnFOIno.ofainFOI)(|nFOI||FOI|)

where {*FOI*}_≠_ is the set of non-redundant residues in position *p* in FOI, (nk) represents the number of combinations of *k* that can be chosen from *n*; and |*FOI*| and |*nFOI*| are the total number of residues in position *p* of FOI and nFOI respectively. As a separate exercise, probability of drawing residues in FOI from the distribution in nFOI with replacement was also considered (S1 Fig) and was found to not improve the performance of the method.

We argue that if *prob_FOI* is sufficiently low, i.e., if *prob_FOI* is smaller than or equal to a threshold value t_s_, then the distribution of residues in FOI is different from that of nFOI and thus the position is differentially conserved (Fig 3G). Thus,

$$prob\_decision(nFOI,p)=\left\{ \begin{array}{ll} \;1, & \;prob\_FOI\leq t_{s}\\ 0, & \;else \end{array}\right.$$

where *prob_decision*(*nFOI*, *p*) is the binary decision of whether the position *p* in FOI is differentially conserved in comparison with the corresponding position in an nFOI; and *t*_*s*_ is the threshold probability (See Determination of threshold values section).

#### Determination of threshold values t_p_, t_e_, t_s_

In the method described above, a binary decision on the uniquely conserved nature of position *p* is made by comparing the FOI and nFOI category measures with a corresponding threshold value (t_p_, t_e_ or t_s_). By altering the threshold value, we tinker with the degree of uniquely conserved nature that passes off as a family-specific site. In order to deliberate the threshold values that result in meaningful delineation of family-specific sites, we used the discriminatory property of the family-specific sites. To this end, we tested several possible values for the threshold, and optimally chose the value that maximally distinguished the families from one another. For instance, the physicochemical threshold t_p_ was systematically tested with values from 0 to 10, at intervals of 1, and the corresponding *pc_scores* were calculated for every FOI, as shown in Fig 2. For a given threshold value, based on the *pc_score* of the FOI, conformity scores were assigned to the sequences in the FOI (*family_scores*) and the rest of the families (*nonfamily_scores*). Conformity scores reflect how well a given sequence conforms to the FOI using proportional weights at the uniquely conserved sites as measured by *pc_score* (See Calculation of Receiver Operating Characteristic in Methods). This process was repeated, considering every family as the FOI, to define an accumulated set of *family_scores* and *nonfamily_scores* for a given value of t_p_. Obviously, the *family_scores* are expected to be reliably higher than the *nonfamily_scores* if the differentially conserved sites are identified accurately. Thus, at the optimal value of t_p_, *pc_score* would best discriminate between the families, and thus between the *family_scores* and *nonfamily_scores*. To quantify this, we calculated the sensitivity, specificity and Receiver Operating Characteristic (ROC) to distinguish the *family_scores* from *nonfamily_scores*. For every t_p_ value, we determined the area under the ROC curve, which is a measure of the ability of *pc_score* to distinguish the *family_scores* from the *nonfamily_scores*, and thus discriminate between the families (S2 Fig). The t_p_ value that yielded the *pc_score* of the best discriminatory capability with maximum area under ROC curve was chosen as the optimal threshold value t_p_.

**Fig 4:**
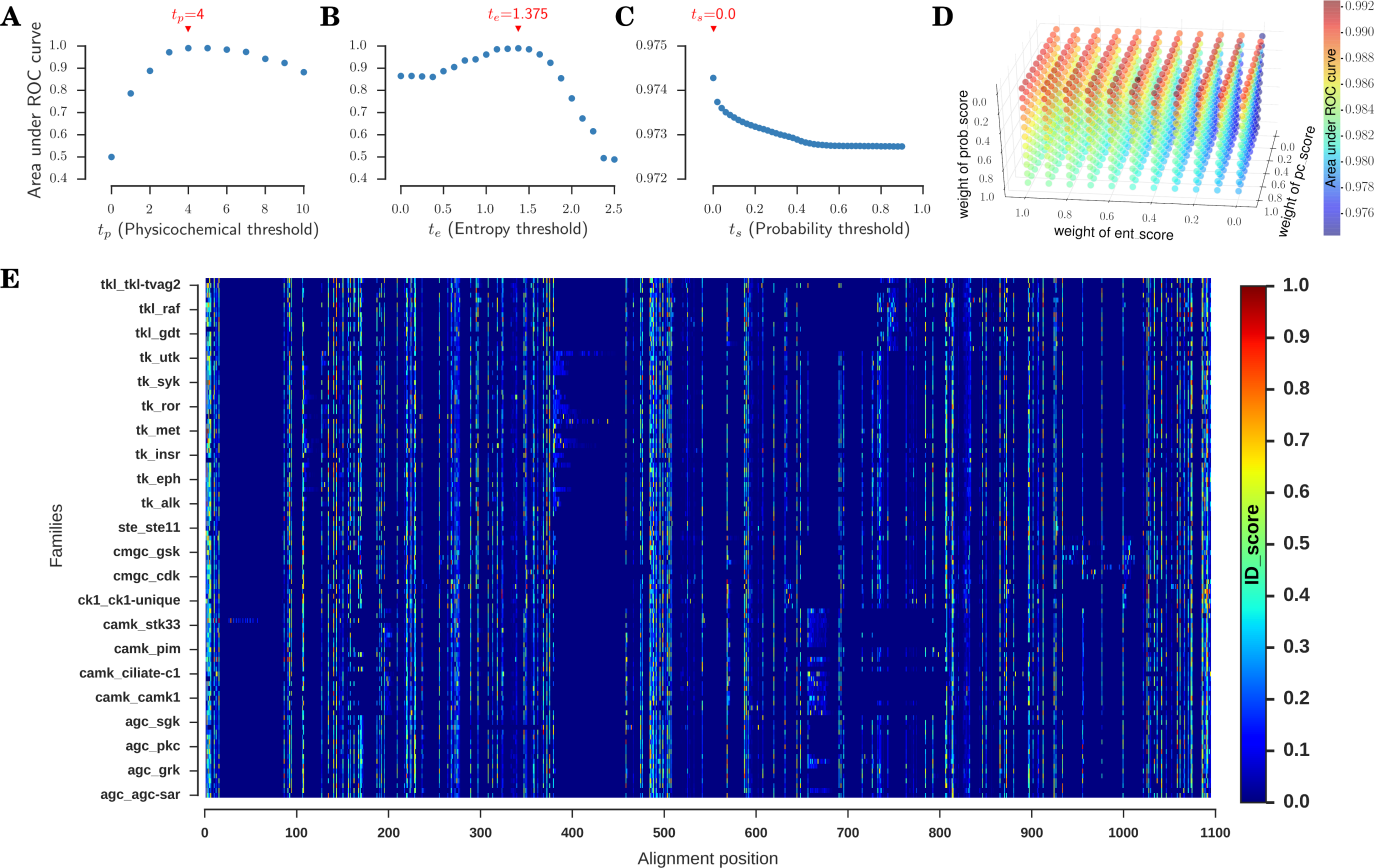
Optimisation of threshold values and integration to a unified ID_score. The thresholds for the 3 measures (*pc*, *ent* and *prob*) were each optimised such that the corresponding scores (*pc_score*, *ent_score* and *prob_score*) had the highest ability to discriminate between kinase families. This was achieved by quantifying how well the *family_scores* were separable from the *nonfamily_scores* at every threshold value in terms of area under the Receiver Operating Characteristic (ROC) curve. The physicochemical threshold t_p_ was systemically tested for all possible values, and the corresponding area under the ROC curve is plotted (A). The maximum area under the curve (0.990) is achieved at a t_p_ value of 4. The area under the ROC curves as a function of entropy threshold t_e_ and probability threshold t_s_ is shown in (B) and (C) respectively. Correspondingly, t_e_ = 1.375 and t_s_ = 0.0 yield the highest area under the curves 0.990 and 0.974 respectively. (D) To optimise the weights for linear combination of the 3 scores (*pc_score*, *ent_score* and *prob_score*), the area under the ROC curve, distinguishing *family_scores* from the *nonfamily_scores*, was calculated for all possible values of *pc_wt* (weight of *pc_score*), *ent_wt* (weight of *ent_score*) and *prob_wt* (weight of *prob_score*) and plotted in a blue-red colour scheme, with *blue* representing the least area and *red* representing the highest. *pc_wt*:*ent_wt*:*prob_wt* of 0.615:0.385:0.0 yielded the highest area under the ROC (0.992). (E) The ID_scores of each of the 107 families as a function of the alignment position is plotted as a heat map in a blue-red scheme. Hotter the colour, higher is the specificity of the site to the family.

Fig 4A shows a plot of the calculated area under the ROC curve for all possible t_p_ values. We find that a t_p_ value of 4 resulted in the highest area under the ROC curve of 0.9904. Thus, if (i) 4 or more physicochemical properties are conserved in FOI and less than 4 physicochemical properties are conserved in nFOI, or (ii) 4 or more physicochemical properties are conserved in FOI and nFOI, but less than 4 physicochemical properties are conserved in FOI+nFOI, the position is decided to be uniquely conserved in FOI with respect to nFOI.

The optimisation procedure described above was followed for the determination of t_e_ and t_s_ values as well. In the case of t_e_, values from 0 to 2.5, at intervals of 0.125, were tested. For each t_e_ value, *ent_score* was calculated and the corresponding area under the ROC curve was determined as described earlier (S3 Fig). Fig 4B shows a plot of the calculated area under the ROC curve as a function of the t_e_ values. It is clear from the plot that the area under the ROC curve peaks to 0.9895 at a t_e_ value of 1.375. In the case of t_s_, values ranging from 0 to 0.9, at intervals of 0.02, were tested (S4 Fig). t_s_ value of 0.0 yielded the highest discriminatory capability (area under ROC curve = 0.9743) to discriminate between the families as seen in Fig 4C.

#### Integrated discriminatory score (ID_score)

The final step in the method is to combine the three measures, *pc_score*, *ent_score* and *prob_score*, and achieve an integrated ID_score. To this end, at every alignment position in a family, we linearly combined the 3 corresponding scores, weighing them appropriately. For an FOI,

$$ID\_score_{p}=(pc\_wt\times pc\_score_{p})+(ent\_wt\times ent\_score_{p})+(prob\_wt\times prob\_score_{p})$$

where *ID_score*_*p*_ is the ID_score at position *p* of the FOI; *pc_score*_*p*_, *ent_score*_*p*_ and *prob_score*_*p*_ are the *pc_score*, *ent_score* and *prob_score* of the FOI at position *p* respectively; and *pc_wt*, *ent_wt* and *prob_wt* are the corresponding weights, which were optimised to provide the highest discrimination between families. Each weight, *pc_wt*, *ent_wt* and *prob_wt*, was independently varied from 0 to 1; and the corresponding ID_score and area under the ROC curve were calculated as described previously. This is to achieve an optimal integration of the 3 measures such that it maximised the capability of the ID_score to discriminate between kinase families. In Fig 4D, we show a 3-dimensional plot with axes for weight of *pc_score* (*pc_wt*), weight of *ent_score* (*ent_wt*) and weight of *prob_score* (*prob_wt*). The area under the ROC curve observed for each combination of weights is plotted in a coloured scheme, with blue indicating the least and red indicating the largest area under the ROC curve. We found that if the three measures, *pc_score*, *ent_score* and *prob_score* are linearly combined with corresponding weights of 0.615, 0.385 and 0.0, the ID_score had the highest discriminatory ability (area under ROC curve = 0.992). It is to be noted that the inclusion of *prob_score* in the method decreased the discriminability of FOI from the rest of the families, and thus the *prob_score* was optimally weighed at 0.0. Thus, we rejected the *prob_score*, and combined the *pc_score* and *ent_score* in a ratio of 0.615:0.385 to achieve the final ID_score.

In summary, through a process of comprehensive measures and rigorous optimisation, we developed a method to score the sites in 107 families of protein kinases that reflects the specific nature of the site to the family (available as S3 File). Fig 4E shows the complete set of ID_scores calculated for all the 107 families at every alignment position. The colour at each position signifies the ID_score in a blue-to-red scheme, with blue indicating the least ID_score and red indicating the highest. As discussed above, ID_score ranges from 0 to 1, where 0 indicates no specificity and 1 indicates high specificity of the site to the family.

### Assessment and validation of ID_score

The ID_score depicted in Fig 4E quantifies the sites based on their differentially conserved nature and the ability to differentiate the family from the other families. Thus, regions of absolute conservation in STY kinases conferring global functions like ATP binding, phosphotransfer catalysis, and overall structural stability of the kinase fold are scored poorly. On the other hand, sites involved in family-specific functions and regulations are likely to be scored favourably. We assessed this by testing the ability of the proposed method to discriminate between the kinase families and identify known family-specific functional and regulation sites.

#### ID_score identified sites cluster sequences into groups better

In Fig 4E, we observe that there are a select few alignment positions which are consistently scored high in most families. In a manner, these positions are possible “specific functional hotspots” that are highly conserved within families, but the nature of conservation differs across families. Due to this property, these positions are hypothesised to have high information content to discriminate families from one another. We tested this hypothesis by first identifying the sites with an ID_score greater than or equal to 0.1 in at least 10% of families. This resulted in 194 alignment positions out of the entire 1094 (S5A Fig).

**Fig 5:**
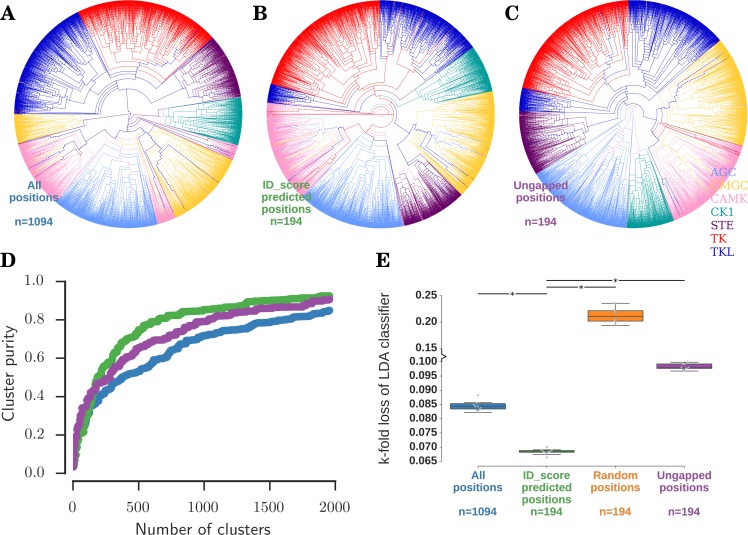
Assessment of the ability of the ID_score to discriminate kinases. Shown are rooted, ultrametric and binary phylogenetic trees of all 5488 kinase sequences, constructed using (A) all 1094 alignment positions, (B) 194 hotspot alignment positions identified by ID_score, and (C) 194 least gapped positions in the alignment as input. The branches in the tree are coloured according to the ‘group’ of the kinase (AGC (*blue*), CMGC (*yellow*), CAMK (*pink*), CK1 (*green*), STE (*purple*), TK (*red*), TKL (*dark blue*)) in the leaf. (D) The family cluster purity for the trees in A-C as a function of number of clusters is shown in *blue*, *green* and *purple* respectively. (E) Upon training and testing a pseudolinear discriminant analysis classifier using all 1094 alignment positions (blue), 194 hotspot alignment positions identified by ID_score (*green*), 194 random positions in the alignment (*orange*) and 194 least gapped positions in the alignment (*purple*), the error in the prediction of the kinase family is plotted. * indicates a *p-value* < 0.001.

For comparative control and analyses, we designed 3 categories containing: (i) all 1094 positions in the master alignment (Fig 4E), (ii) 194 hotspot positions, as identified by ID_score (S5A Fig), and (iii) 194 positions with the least number of gaps in the alignment (S5B Fig). In the first category, using all the 1094 positions in the alignment as input, we constructed a phylogenetic tree [77] (See Phylogenetic tree in Methods) of all the 5488 sequences in the dataset. In Fig 5A, the tree is illustrated with branches colour-coded according to the KinBase [62] ‘group’ of the leaf kinase. It can be appreciated from the tree that although complete information available in the sequences is used as an input, kinases of group CAMK (*pink*) and CMGC (*yellow*) are split into 3 and 2 separate clusters respectively.

In the second category, using only the 194 alignment positions identified by ID_score, we constructed a similar phylogenetic tree of all the 5488 sequences (Fig 5B). In theory, since only a fraction of information is used as input to the tree, we expect the tree to be more error-prone in comparison to Fig 5A. On the contrary, we observe good clustering of all the groups except TKL (Fig 5B, *dark blue*). This is possibly due to successful filtering of the noisy and indiscriminate sites, and use of only the most informative and discriminative positions. Upon closer examination, we find that the smaller cluster of TKL (Fig 5B, *dark blue*), separated from the primary TKL cluster, is predominantly composed of sequences of the STKR (Serine Threonine Kinase Receptor) family (S5C Fig). This is indeed interesting because although classified within the Tyrosine Kinase–Like (TKL) group, STKRs are the only receptor kinases that phosphorylate Ser / Thr residues on the substrates and thus have the properties of both the Tyr phosphorylating kinases (TK and TKL) and Ser / Thr phosphorylating kinases. The tree built using ID_score identified sites correctly captures this by placing the STKR family between CAMK (*pink*) and TK (*red*).

In the third category, we used 194 least gapped positions from the alignment to draw a phylogenetic tree of 5488 sequences (Fig 5C). As can be noticed, the tree is highly similar to the one constructed using the ID_score predicted sites, with good clustering of all groups except TKLs (S5D Fig). Are ID_scores merely identifying the ungapped positions in the alignment, and therefore not useful? Or, do ID_scores identify sites which contain only as much information as contained in ungapped positions? Are the trees constructed in Fig 2B and 2C truly similar to each other? To answer these questions, we probed the trees at a finer level and analysed the cluster purity in the 3 categories at the level of families, as described below.

#### ID_score identified sites cluster sequences into families better

We cut the phylogenetic trees (Fig 5A–5C) at different branch lengths from the root. At every cut, we counted the number of clusters obtained and calculated their purity at the level of ‘families’ (See Cluster purity calculation in Methods). We note that upon cutting the tree at increasing branch lengths from the root, the number of resulting clusters as well as the cluster purity increases. If cut at the extreme terminal of the tree, there would be as many clusters as there are number of sequences, and the purity of the clusters would be 1. In Fig 5D, we plotted the family cluster purity of the trees constructed using all positions, ID_score predicted positions and ungapped positions (Fig 5A–5C) as a function of the number of clusters in *blue*, *green* and *purple* respectively. It is clear from the plot that when the ID_score identified sites are alone used for the construction of a phylogenetic tree, the family clusters are purer (*green*) than when all the sites (*blue*) or an equal number of ungapped positions (*purple*) are used. We conclude that although the ID_score identified sites seemingly performed only as well as ungapped sites in clustering the kinases into groups (Fig 5B and 5C), they outperform the ungapped positions in clustering the kinases more finely into families (Fig 5D). Thus, we interpret that ID_score does not trivially pick the ungapped positions but successfully identifies sites that efficiently discriminate the kinases into groups and families.

#### ID_scores help classifiers predict the families of sequences

As interpreted from the previous analyses, if ID_score could indeed identify sites that contain information to discriminate between the families, we hypothesised that a simple Linear Discriminant Analysis (LDA) classifier would be able to predict the families of the kinase sequences better if trained with ID_score identified sites. To this end, we trained a pseudolinear classifier (See Classifier analysis in Methods) with the family associations of a random 90% of the sequences in the dataset. The classifier was then tested on the remaining unseen 10% of the sequences. This hold-out training and testing was repeated 10 times. Upon using all the 1094 alignment positions for training and testing, the error rate for the prediction of the family is about 8.5% (Fig 5E, *blue*). However, when only the 194 sites identified by the ID_score were used for training and testing, the error rate dropped to 6.7% (Fig 5E, *green*). This improvement in accuracy is in spite of lesser information as input (194 alignment positions as opposed to 1094 positions leads to 82% decrease in information) which should theoretically decrease the performance of the classifier. This can be seen in the *orange boxplot* of Fig 5E, which shows the error rate (~20%) of the classifier trained and tested on 194 random positions in the alignment. Also, an equal number of least gapped positions in the alignment, when used to train and test the classifier, made it perform poorly (Fig 5E, *purple*). Taken together, our analyses strongly suggest that ID_score method has successfully scored the sites in a manner that not only maximises the discriminability across families, thus increasing the efficiency of prediction of family, but also clusters the sequences into groups and families in an accurate and meaningful way.

#### Identification of family-specific protein-protein interaction sites: A case study with CDK

**Fig 6:**
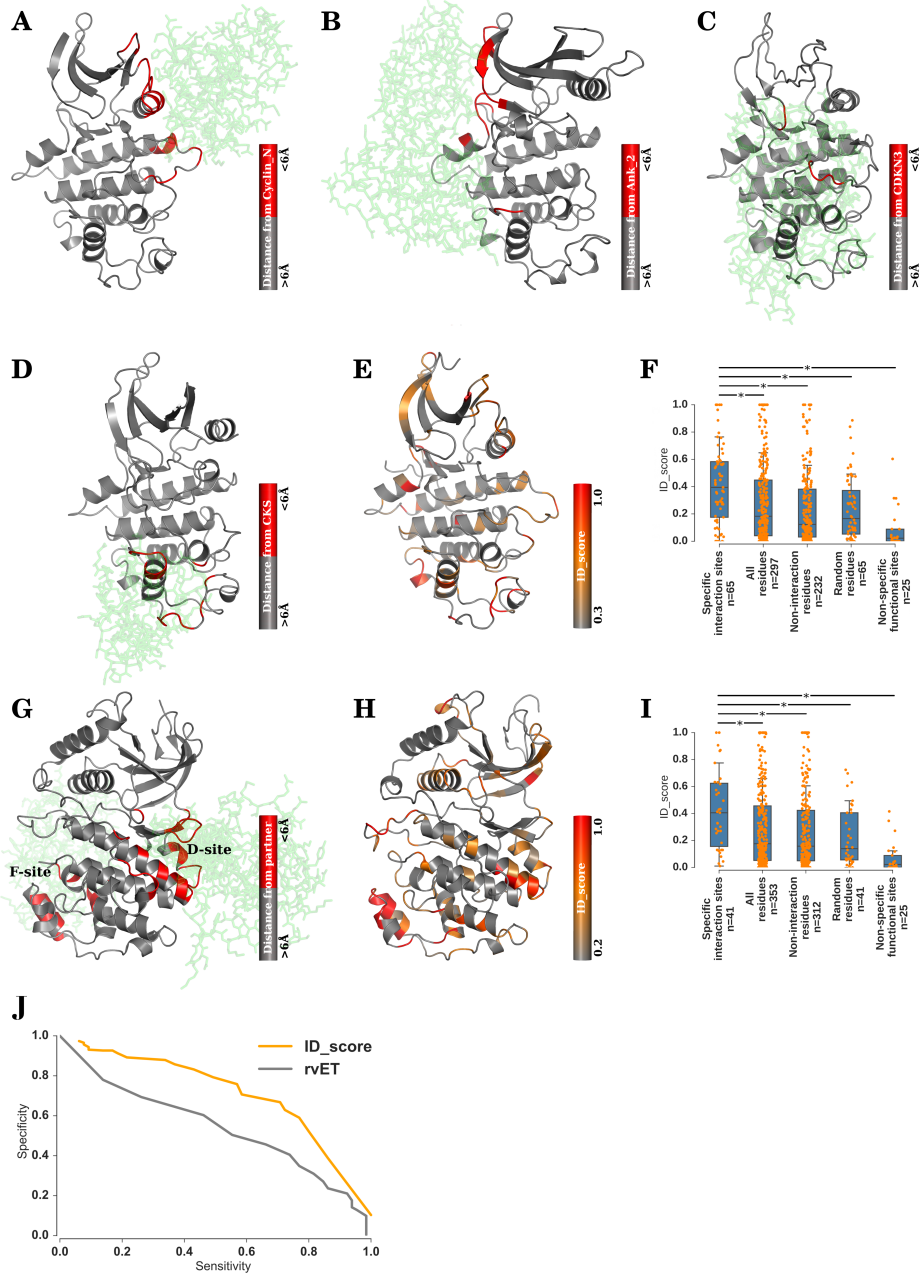
ID_score identifies sites of family-specific functions. Family-specific interactions experimentally found in CDK family of kinases are illustrated in A-D. The CDKs are shown in cartoon and the interacting partners cyclin_N (A), Ankhyrin_2 (B), CDKN3 (C) and CKS (D) are shown in green sticks representation. The residues in kinases are coloured *red* or *gray* depending on whether or not they form contact interface with the binding partner respectively. (E) CDK is represented in cartoon, and the individual residues are colour coded in a gray-red scheme depending on the ID_score of the sites, with *red* representing high ID_score and *gray* representing low ID_score. (F) The distribution of ID_scores of the known specific interaction sites is compared with that of all the residues, non-interaction sites, random sites and nonspecific functional sites in CDK. It is seen that the specific interaction sites are scored significantly higher by the ID_score than the other comparisons. (G) Residues experimentally known to be involved in cognate substrate (*green sticks*) recognition in MAPK (cartoon) is shown in *red*. (H) MAPK is illustrated in cartoon representation, with individual residues coloured according to their ID_scores. (I) The ID_scores of specific substrate interaction sites are significantly higher than that of all residues, non-interaction sites, random sites and nonspecific functional sites in MAPK. (J) The performance of ID_score method (*orange*) in detecting the family-specific functional sites in CDK and MAPK, in terms of sensitivity and specificity, is plotted along with that of real-value evolutionary trace (rvET, *gray*) method. * indicates a *p-value* < 0.001.

We have so far demonstrated the ability of the proposed method to identify sites that efficiently differentiates the families of kinases. However, a more meaningful and biologically relevant validation of the method is to check if the method identifies the known family-specific functional sites. For case study, the widely studied CDK family of kinases was chosen. Upon analysing iPfam [78], the database of all domain-domain interactions in protein crystal structures, we identified 4 CDK-specific interacting partners: Cyclin_N (Pfam ID: PF00134), Ank_2 (PF12796), CDKN3 (PF05706) and CKS (PF01111). It is known through several experimental studies and structure determination that these interactions are specific to kinases of CDK family and are unknown to occur in other families of STY kinases. We have illustrated the structural complexes of these interactions in Fig 6A–6D, where CDK is represented in cartoon and the interacting partner is shown in *green sticks*. The residues in CDK are coloured *red* or *gray* depending respectively on whether or not they interact with the partner in the complex. The distance criterion for interaction is considered as 6 Å. In Fig 6E, cartoon representation of CDK, whose residues are coloured in a gray-to-red colour scheme based on their ID_score, is depicted. We observe that the sites known to be involved in family-specific protein-protein interactions (Fig 6A–6D, *red*) are indeed scored high by the proposed method (Fig 6E, *orange* and *red*). As seen in Fig 6F, the residues involved in family-specific interactions have ID_scores significantly higher than all residues (*p-value* < 0.001), non-interaction residues, random residues and non-specific functional sites (ATP binding loop, catalytic loop, salt bridge residues, DFG and APE motifs). It should be noted that not all the interacting residues, as identified by the distance criterion from the crystal structures, may contribute equally towards the interaction or binding energy. Vice versa, not all residues with high ID_scores ought to be involved in a specific protein-protein interaction. Some of them may play roles in other family-specific functions / regulations. However, we quantified the same and conclude that, as a general trend, the proposed method identifies sites involved in family-specific protein-protein interactions.

#### Identification of family-specific substrate recognition sites: A case study with MAPK

MAPK is a family of CMGC group of STY kinases, whose substrate recognition sites and motifs are well studied. It is known through several biochemical and structural studies that MAPK stabilises the kinase-substrate complex through interactions at distal docking sites in the kinase domain [79–83]. These distal sites are away from the kinase active site, and recognise specific sites on the cognate substrates. Two such docking sites, D-site and F-site, predominantly dictate the substrate specificity of MAPKs (Fig 6G), apart from the P+1 site. Consolidated from several crystal structures of MAPK complexes with substrate peptides and mimics, we mapped the D-site and F-site residues that interact with substrates (Fig 6G, *red*). Fig 6H shows the MAPK fold in which the residues are colour coded in a gray-to-red scheme based on the ID_score of the sites. It can be seen that the sites experimentally known to recognise substrates in MAPK (Fig 6G, *red*) are scored high by the current method (Fig 6H, *orange-red*) as well. This excellent agreement between the known substrate recognition sites and ID_score identified family-specific sites is quantified in Fig 6I, where the substrate recognition sites have significantly higher ID_scores than the other sites in comparison (*p-value* < 0.001).

The ability of our method to identify the family-specific functional sites in CDK and MAPK, defined from X-ray crystal structures (Fig 6A–6D and 6G, *red*), was then compared with that of a previously published method referred to as real-value evolutionary trace (rvET) [44]. rvET is a hybrid method that combines entropy and phylogeny information, further drawing from experimental structures. The phylogeny aspect in rvET can be adjusted to cluster the nodes at different distances from the root, thereby making it possible to isolate kinase families from one another at suitable thresholds. The sensitivity and specificity of identifying the family-specific functional sites were calculated at a series of score thresholds and plotted in Fig 6J. From the plot, it can be appreciated that ID_score identifies the family-specific functional sites with better accuracy than rvET.

#### Identification of family-specific oncogenic driver mutation sites

**Fig 7:**
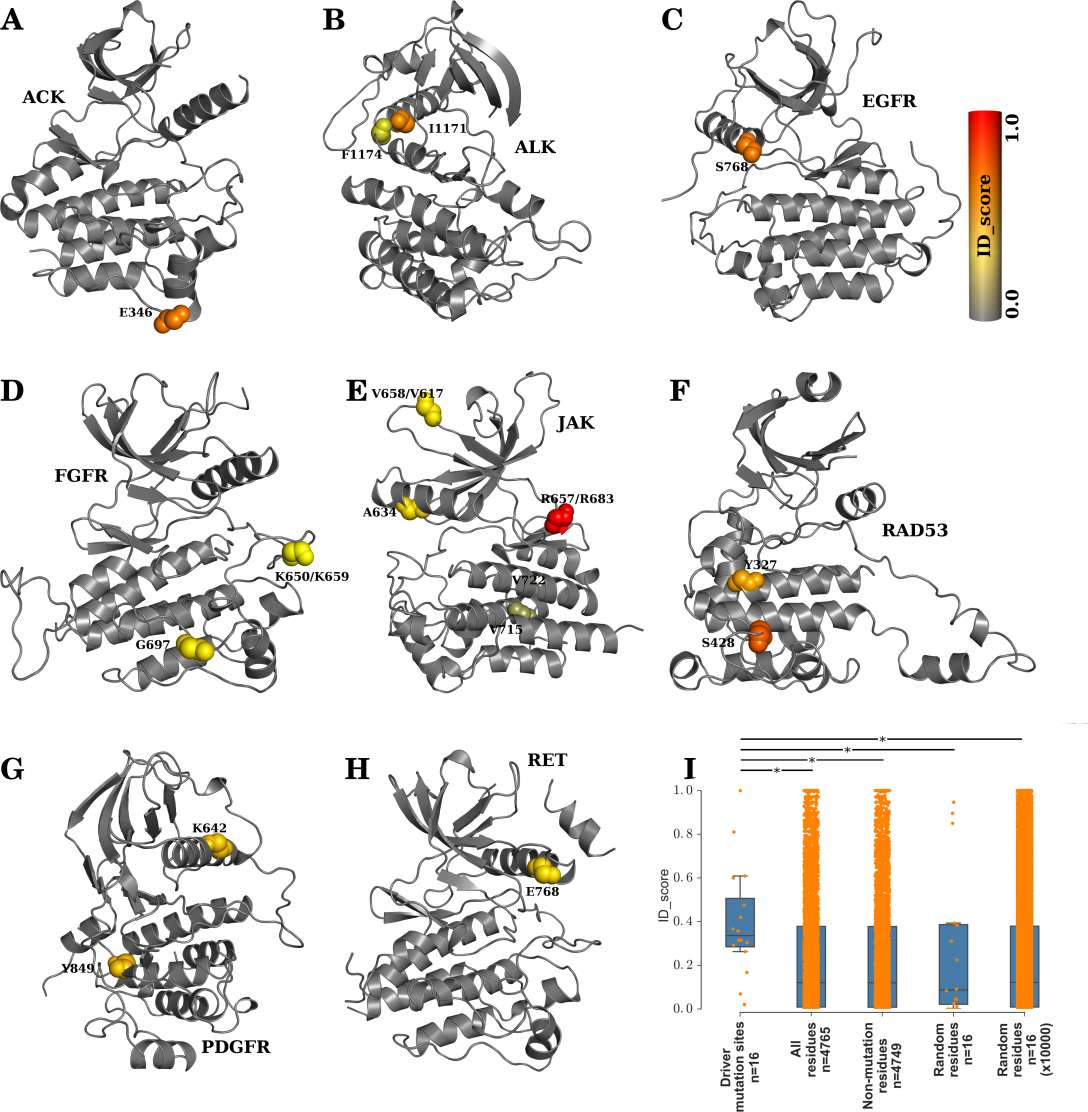
ID_score identifies family-specific oncogenic driver mutation sites. Driver mutation sites that are specific to ACK, ALK, EGFR, FGFR, JAK, RAD53, PDGFR and RET (A-H) are represented as spheres in the corresponding kinase folds, and coloured according to their ID_scores. (I) It is seen that the specific mutation sites are scored significantly high by the ID_score method, when compared to all residues, non-mutation residues and random residues in the families. * indicates a *p-value* < 0.001.

Being implicated in numerous cancers, STY kinases have an extensive literature on the cancer causing mutation sites [84,85]. We hypothesised that if a mutation disrupts the activity of the kinase by activating or inactivating it, by direct or indirect means, the residue ought to have been crucial for the functionality of the kinase. In other words, the cancer causing mutation sites, if family-specific, are most likely family-specific functional sites and thus will be identified by the present method. From KinDriver [86], a database of all known cancer causing driver mutations in kinases, we identified the missense mutation sites that were recorded only in a specific family of kinases with a relative frequency of >2. The identified family-specific cancer causing driver mutation sites are represented as spheres in Fig 7A–7H in families ACK, ALK, EGFR, FGFR, JAK, RAD53, PDGFR and RET respectively. The mutation sites, as represented by spheres, are coloured in a gray-to-red scheme, representing the ID_score of the site. We can appreciate that most of the sites are scored highly by the ID_score method and are thus represented in the *orange-red* range. Quantitatively, we find that the known family-specific driver mutation sites were scored high by the ID_score method (Fig 7I) when compared to other residues.

### Application

So far, we have discussed the development of the ID_score method and its successful identification of known family-specific functional sites in various families. In this section, we present the application of the method in falsifiable prediction of family-specific functional sites for all known protein kinase families and discuss the predictions made for PKG and PKC families.

#### Prediction of family-specific protein-protein interaction sites: A case study with PKG and PKC

**Fig 8:**
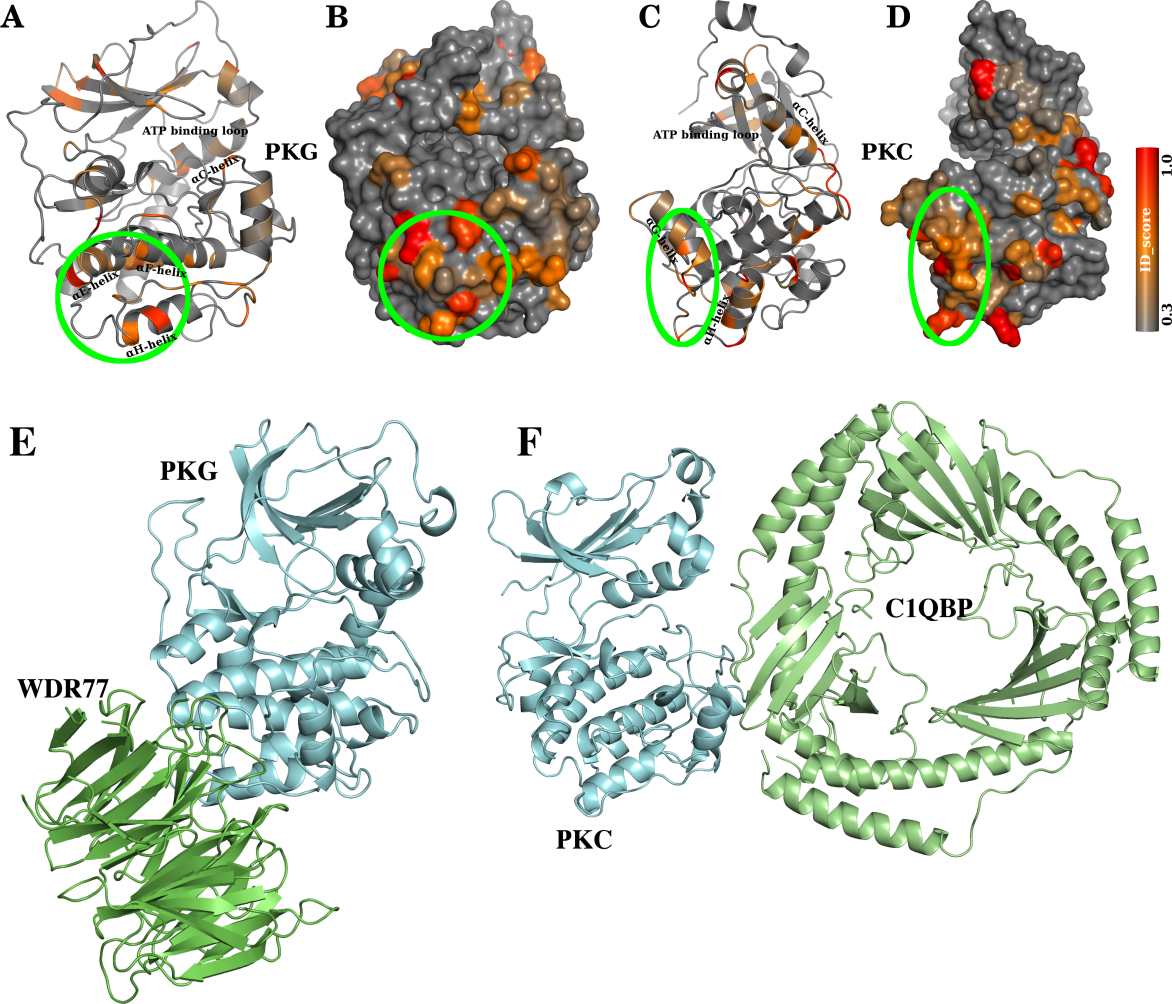
Testable predictions of specific protein-protein interaction sites. PKG kinase is shown in cartoon (A) and surface (B) representations, with the residues coloured according to their ID_scores. A cluster of high ID_scored residues that are solvent accessible and spatially proximal to each other is marked (A-B, *green circle*). This region that is formed at the junction of αE, αF and αH-helices is a putative candidate region involved in specific protein-protein interaction, potentially with WDR77. PKC kinase is shown in cartoon (C) and surface (D) representations, with the residues coloured according to their ID_scores. A cluster of high ID_scored residues that are solvent accessible and spatially proximal to each other is marked (C-D, *green circle*). This region that is formed at the junction of αG and αH-helices is a putative candidate region involved in specific protein-protein interaction, potentially with C1QBP. Models of (E) PKG-WDR77 and (F) PKC-C1QBP complexes docked at the ID_score predicted interfaces are presented.

We parsed BioGrid [87], a database of all experimentally known domain-domain interactions, and identified all interactions of kinases that were verified by at least two different techniques. From this highly confident set, we identified that the WD Repeat domain 77 (WDR77) specifically interacts with the kinases of the PKG family. Although several studies have shown the interaction [88], the site of interaction in the kinase domain is unknown. Cartoon and surface representations of a kinase fold is depicted in Fig 8A and 8B respectively, in which the residues are coloured in a gray-to-red scheme based on the ID_score of the sites. We observe a cluster of solvent accessible, high ID_score sites in close proximity to each other at the conjunction of αE, αF and αH helices (Fig 8A and 8B, *green circle*). We predict that this region is the potential WDR77 interaction region in PKG. Likewise, we also identified that C1QBP (Complement 1, Q Subcomponent Binding Protein) specifically interacts with kinases of PKC family [89]. As can be seen in the ID_score-colour-coded cartoon and surface representations of PKC, a cluster of family-specific functional sites are seen around the αG helix (Fig 8C and 8D, *green circle*). We predict that αG helix is involved in the binding of PKC kinases with C1QBP. For the purpose of visualisation, we illustrate the PKG-WDR77 (Fig 8E) and PKC-C1QBP (Fig 8F) complex models docked at the interface predicted by ID_score.

#### Prediction of sites of functional specialisation in all known kinases

Encouraged by the ability of the method to identify accurately the functionally specialised residues and cancer driver mutation sites in some of the well-studied kinase families, we present the predicted sites of functional specialisation in all the 107 kinase families used in the study (predicted sites are documented in the S3 File). Each site in every family is given a score that ranges between 0 and 1, where 0 indicates no functional specificity and 1 indicates high functional specificity. We emphasise that our method identifies sites of functional specialisation in a family and not global functional regions like catalytic loop and ATP binding site.

To our knowledge, this is the first and only available resource that provides the functional sites in a large scale to the kinase community. We hope that this serves as a useful starting point for biochemical and structural studies as well as *insilico* drug targeting studies. The users are encouraged to set arbitrary cut-off values of the ID_score to identify the sites to pursue. For instance, for an uncharacterised kinase family under study, the users may set a cut-off of, say, 0.4 and restrict their preliminary analyses to sites with an ID_score of ≥ 0.4 in the family. This lets the user work with a tangible set of residues, which may further be guided by other known features of the kinase. The current study also opens up a consequent scope to find the functional implications of the identified sites. As already noted, the sites predicted by our method may impart functional specialisation to the kinase by way of specific substrate recognition, protein-protein interaction or allostery.

## Discussion

An emerging picture of signalling networks strongly suggests a complex system of organisation with regulatory orchestration at multiple levels [90]. Traditionally, this complex scheme of control has been studied in a reductionist approach, attributing modularity to the interacting proteins, associated domains, scaffold proteins, and hubs in the network [91–94]. If we extend the theory of modularity and reductionism to within a domain, we expect division of the multitude of specific functions of the domain to certain subparts, conventionally known as functional motifs. Supporting evidence of this theory includes alteration and loss of specific function upon mutation of certain residues. Additionally, regardless of whether function of a protein is modular or emergent, it is well known that subtle functional differences between proteins reside in a few key residues.

On an average, an STY kinase catalytic domain is 250 residues long. Within this region exists information for (i) global attributes like stable structure formation and catalysis of phosphotransfer, and (ii) specific attributes like recognition / phosphorylation of its cognate substrate, interactions with specific binding partners and response to specific signal. Identification of functional sites / motifs conferring global attributes is feasible through experimental mutagenesis studies and *in silico* detection of conserved sequence patterns across all kinases. For instance, global functions like ATP binding and catalysis are attributed to the GXGXXG and HRDLXXXN motifs respectively. On the other hand, specific functional attributes are not only challenging to detect using experimental methods, but also difficult to identify *in silico*. In the present study, we have identified the family-specific functional sites in all known families of eukaryotic protein kinases.

Previous studies that attempted to identify differentially conserved family-specific functional sites relied heavily on the measurement of a single property and lacked an integrated approach. In the present study, we have for the first time used physicochemical, entropy and probability measures to identify family-specific functional sites in the most meaningful manner. The *pc_score*, which scores a position based on the conservation of physicochemical properties, tolerates an Arg to Lys substitution more than an Arg to Leu substitution. The *ent_score*, on the other hand, tolerates certain other substitutions better, and detects conservation of a position that could be missed out by *pc_score*. For instance, if a position in an FOI is populated with 2 physicochemically dissimilar amino acids, and the equivalent position in nFOI is populated with 6 different hydrophobic amino acids, *pc_score* would disregard the position, but *ent_score* classifies it as differentially conserved in FOI. During the course of the study, many metrics including mutual information, that measures correlated mutations between residue positions, were considered to score the uniqueness of sites. We found that the area under the ROC for the mutual information score was not better than that obtained through the individual Shannon entropy (*ent_score*). This is because, unlike methods that stringently mandate absolute conservation of an amino acid or physicochemical property, *ent_score* allows for limited number of substitutions at a residue position, thereby not punishing the correlated mutation sites. Although the correlations between residue positions is unidentified by our method, the individual sites harbouring correlated mutations are still scored high by the ID_score. This is evident by the fact that ID_score performs better than the rvET method, which considers covariances.

The third measure, *prob_score*, calculates the probability that the exact set of amino acids at a position in FOI can be drawn from a pool of amino acids in nFOI. This is a highly stringent score that punishes dissimilarity heavily and gives a high score only if the amino acids in the FOI and nFOI are similar in composition. One could imagine why such a stringent measure failed to add to the discriminability across kinases in the integrated method. Skew and lack of variance in the distribution of *prob_scores* made the discriminability between FOI and the rest of the families poorer. As an extreme example, the absence in nFOI of one of the amino acid residue types found in FOI will directly lead to a poor score even if the other amino acids residue type distributions matched well.

We note that ID_score primarily uses the multiple sequence alignment and prior classification of sequences into families as input to calculate the specificity determinants. As a result, error in the alignment, especially in cases of large and diverse superfamilies with sequences less than 20% sequence identity, can affect the accuracy of the method. Similarly, incorrect classification and subgrouping will also propagate through the method and result in reduced accuracy.

A couple of previous studies have looked at group-specific functional signatures and specificity determinants in kinases [60,95]. For the purpose of the current study, we intended to understand the family-specific functional sites in kinases, which will be of immense value to suppress drug cross-reactivity. Using the same method to identify group-specific functional sites is an interesting proposition that warrants a separate study by itself. Group-specific features like C-terminal tail binding of AGC-group kinases and SH2 domain interaction of TK-group kinases may be predicted if ID_score is used at the group-level.

After identifying the family-specific sites in all the 107 families of kinases by maximising the discriminability across families, we might ask if all the identified sites are indeed functional. Although, we have addressed the question by showing excellent agreement between the ID_scores and known family-specific functional sites, answering the question in its entirety is difficult. Nevertheless, it is advantageous to know the bare essential set of sites that renders PKA different from, say, Src. In fact, if needed, pair wise comparisons of families can be easily extracted from the ID_score method, and the specific set of residues that differ between the two families can be identified. This leads us to an interesting thought experiment: if we replaced the bare essential sites in PKA that differentiates a PKA from Src with those of Src, would we expect the PKA to inherit some of the properties of Src kinase? If the modularity of functionality within the kinase domain is valid and we have identified the family-specific functional sites, including the redundant ones, one should expect so. Extending this thought experiment, if one were to identify the functions of each of the family-specific sites identified by the method, would it be possible to build a synthetic protein kinase with customised functions?

Although the literature is ripe with studies of cancer causing mutations in kinases [85], mechanistic / functional reasoning behind why a mutation derails the functionality of a kinase is understood more in retrospect than in advance. This is because a complex network of interactions and abundant redundancy in the protein’s functionality makes it an extremely challenging task. In this study, we show that those cancer causing mutation sites, which occur within specific families, are identified with high scores by the ID_method. Furthermore, we also show biologically relevant clustering of kinase families, when aided by ID_score method. Taken together, our analyses suggest that ID_score can successfully predict the family-specific functional sites. Using ID_scores, we predict the interaction sites in PKG and PKC for binding WDR77 and C1QBP respectively. Although developed and demonstrated for the STY kinase superfamily, the method is inherently transferable to other protein superfamilies, and is expected to aid identification of functional sites and characterisation of ambiguous / new families.

In summary, we curated an unbiased and non-redundant dataset of 5488 sequences of kinase catalytic domains from diverse phyla belonging to 107 families of 7 distinct groups. After careful alignment of all the curated sequences, we developed an integrated approach to detect differential conservation of residues in kinase families. Using measurements of physicochemical properties, Shannon entropy and probability, we scored the selectively conserved nature of the all the sites in the kinase families. Furthermore, by maximising the discrimination across families, we succeeded in optimising the threshold criterion for each method that passes a differentially conserved position as a family-specific functional site. Finally, we integrated the 3 scores to attain a unified ID_score that scores the sites in kinase families depending on their functional specificity, characteristic to the family. We have assessed and validated the ability of ID_score to (i) discriminate and cluster the kinase families in a meaningful way and (ii) identify family-specific functional sites. Further, we demonstrate the application of the method in prediction of protein-interaction sites. Taken together, we developed an integrated method and successfully identified the family-specific functional sites in all known eukaryotic kinases.

## Methods

### Dataset

After UniProt_ID-family mapping was established (Fig 1C), the kinase catalytic domains were to be excised from the full length sequences. To this end, families in which information on kinase domain boundary [70] is not known for any sequence were eliminated. Then, within every family, the sequence with the most number of experimentally solved structures was identified as the most well studied STY kinase of the family, or the template sequence of the family. In case of absence of solved crystal structures for a family, a sequence in which the kinase domain boundary is known was randomly chosen as the template sequence of the family. The sequences within every family were multiply aligned [71] and the kinase catalytic domains were extracted from the sequences by mapping the topologically equivalent residues corresponding to the kinase catalytic domain of the template sequence. Thus derived kinase catalytic domain sequences of a family were further filtered to contain residue length in the range of 150 to 350 residues and clustered at 90% sequence identity [72] to remove redundancy and bias in the dataset. After this step, if a family contained less than 5 kinase catalytic domain sequences, it was discarded; and if a family contained more than 200 sequences, it was clustered at progressively higher sequence identity thresholds until it contained less than 200 sequences. In total, clustering threshold of 90% sequence identity resulted in more than 200 sequences in only 6 of the 107 families. The families and their threshold identities are MAPK (80%), STE20 (80%), MLK (80%), CDK (70%), IRAK (60%) and CAMKL (50%). Since only less than 6% of the kinase families were clustered at lower that 90% sequence identity and such families still retained an average of 184 sequences per family, the contribution of error due to this is considered minimal. This procedure was repeated for every family and the kinase domain sequence-family mapping was established (See S1 Text in Supplementary Information and Fig 1D).

### Alignment

An accurate multiple sequence alignment (MSA) of the catalytic domain sequences in the dataset is a prerequisite to probe the family-specific functional sites in the sequences. However, given the large divergence in the dataset, precise error-free alignment of all the sequences in a single MSA is challenging. The approach used in the study is to first align [71,96] the sequences within families and obtain a consensus sequence / profile for each family [97]. Subsequently, the consensus sequences of all families within a group were multiply aligned using sequence and structure information. This crucial step was feasible because reliable hierarchical classification of STY kinases into groups / family and crystal structures in active conformations belonging to different families within a group were available (See list of PDB IDs and hierarchy used in S6 Fig). Superposition of the crystal structures, with not more than one structure per family, if available, was used to guide the alignment of profiles of families within groups. This resulted in profile or consensus sequence for each group, which were then multiply aligned using sequence and structure information. Again, superposition of structures, with not more than one structure per group, if available, guided the alignment of across-group profiles, resulting in a profile / consensus sequence for the entire STY kinase dataset. The above described method for hierarchical alignment of profiles to arrive at a consensus sequence for STY kinases was implemented using a python script called Fammer [98]. Considering the consensus sequence of the STY kinase as the template, all the sequences in the dataset were aligned to it in a statistical method [99] to get the final MSA as described in previous studies [52]. During this procedure, a few families / sequences that did not align well within themselves or the entirety of the kinase database were manually eliminated. The final alignment consisted of 5488 STY kinase domain sequences of 107 families.

### Calculation of Receiver Operating Characteristic (ROC)

For a threshold value, say, t_p_, the corresponding *pc_score* was calculated as described in the Results section. For every FOI, *pc_score* is a vector of length N, where N is the length of alignment positions, containing values in the range of 0 and 1 with 0 representing no family-specificity of the position and 1 representing maximum family-specificity. All sequences in the database are given a conformity score with respect to the *pc_score* of a family of interest (FOI), say f1. The conformity score of a sequence is the sum of all *pc_score* values at those positions in which the sequence conforms to the sequences of the family f1. A sequence is considered to conform to f1 sequences at position *p* if the amino acid in the sequence at *p* is one of those that populate the position in the sequences of f1. For instance, if at an alignment position *p*, where, say, *pc_score* of FOI f1 at *p* = 0.4, sequences in f1 are populated with A, L, I and M, and a sequence to be conformity scored contains I, then the conformity score of the sequence is increased by 0.4. On the other hand, if the sequence to be conformity scored contains V at *p*, then, conformity score is unchanged.

$$conformit{y\_score}_{\left(s,FOI\right)}=\sum_{p=1}^{N}{pc\_score}_{FOI,p}[s_{p}\in {\left\{{FOI}_{p}\right\}}_{\neq }]$$

where *s* is the sequence for which score is to be evaluated according to its conformity to FOI; N is the total number of positions in the alignment; *pc_score*_*FOI*,*p*_ is the *pc_score* of FOI at position *p*; and [*s*_*p*_ ∈ {*FOI*_*p*_}_≠_] is the conditional clause as to whether the amino acid at position *p* in *s* belongs to a set of non-redundant amino acids in position *p* of FOI.

In this manner, each of the 5488 sequences in the dataset is each given a conformity score with respect to the *pc_score* of FOI f1. These conformity scores are divided into 2 categories: (i) *family_scores*, when *s* is a sequence of the FOI and (ii) *nonfamily_scores*, when *s* is a sequence of an nFOI. This process is repeated, considering each family (f1, f2, …, f107) as FOI, and the *family_scores* and *nonfamily_scores* are augmented.

Similarly, for optimisation of the entropy threshold t_e_,

$${conformity\_score}_{s,FOI}=\sum_{p=1}^{N}{ent\_score}_{FOI,p}[s_{p}\in {\left\{{FOI}_{p}\right\}}_{\neq }]$$

and probability threshold t_s_,

$${conformity\_score}_{s,FOI}=\sum_{p=1}^{N}{prob\_score}_{FOI,p}[s_{p}\in {\left\{{FOI}_{p}\right\}}_{\neq }]$$

were carried out.

The set of *family_scores* is expected to be reliably higher than the *nonfamily_scores* upon accurate determination of the threshold value. Thus, we calculated the sensitivity, specificity and the ROC for the two scores. In essence, the area under the ROC curve implies the discriminability between families based on the *pc_score* (*ent_score* or *prob_score*) derived using a specific threshold t_p_ (t_e_ or t_s_).

### Phylogenetic tree

A phylogenetic tree was constructed by considering all the 1094 positions of the master sequence alignment using FastTree [77]. This resulting tree was mid-point rooted and made ultrametric by extension of all the terminal branches to a constant distance from root. Finally, if the tree had multiple originating branches at any given node, it was bifurcated and thus converted to a binary tree [100]. This tree is shown as a circular cladogram in Fig 5A. The same protocol was used for the construction of trees in Fig 5B and 5C, with the exception that specific chosen positions (as identified by ID_score and those containing the least number of gaps respectively) of the alignment were used as input to for the construction of the tree.

### Cluster purity calculation

The phylogenetic tree that is rooted, ultrametric and binary was cut at different distances from the root resulting in different number of clusters. For each cut, cluster purity of the resulting clusters was calculated as follows:

$$cluster\_purity=\frac{1}{n}\sum_{i=1}^{k}{max}_{j}|c_{i}\cap f_{j}|$$

where n is the total number of leaf sequences; k is the number of clusters generated; *c*_*i*_ is the set of leaf sequences in the i^th^ cluster; *f*_*j*_ is the family classification that has the maximum number of leaves in cluster *c*_*i*_.

### Classifier analysis

We trained and tested a simple pseudolinear classifier to understand its ability to ascertain family classification to a kinase sequence. In a hold-out approach, we used a random 90% of the sequences to train the classifier and the remaining 10% to test, repeating 10 times. The sequences were in aligned format and the corresponding family associations of the sequences were used to train the classifier. The k-fold loss in the performance of the classifier in the test set was quantified [101]. The classifiers were trained and tested using all the alignment positions (Fig 5E, *blue*), positions identified by the ID_score (Fig 5E, *green*) or the positions with the least number of gaps (Fig 5E, *purple*). This analysis was performed using the Statistics and Machine Learning toolbox of MATLAB [102].

## Supporting information

S1 File UniProt_ID-to-family mapping of 34,881 kinase sequences into 164 families. (XLS)

S2 File Multiple sequence alignment of kinase domains of 5488 sequences from 107 families of eukaryotic protein kinases. (TXT)

S3 File Position-wise ID_scores for each of the 107 kinase families used in the study. (XLS)

S1 Text Constraints used in dataset selection. (DOCX)

S1 Fig Use of random chance of amino acid retrieval with replacement. The threshold for the *prob* measure was optimised such that the corresponding *prob_score* had the highest ability to discriminate between kinase families. This was achieved by quantifying how well the *family_scores* were separable from the *nonfamily_scores* at every threshold value in terms of area under the Receiver Operating Characteristic (ROC) curve. *t*_*s*_, the threshold probability of obtaining the exact set of amino acids in position *p* in FOI when one repeatedly draws, with replacement, from the set of amino acids in the same position of nFOI was systemically tested for all possible values, and the corresponding area under the ROC curve is plotted. The maximum area under the curve (0.970) is achieved at a *t*_*s*_ value of 0.02. This is lower than that of the *t*_*s*_ calculated without replacement.
(TIFF)

S2 Fig Receiver Operating Characteristic quantification for discriminability between family_scores and nonfamily_scores at different *t*_*p*_. For *t*_*p*_ values ranging from 0 to 10 (A-K), the *family_scores* (*blue*) and *nonfamily_scores* (*red*), augmented across families, are shown as normalised histograms; and the corresponding areas under the ROC are indicated. *t*_*p*_ value of 4 (E) yielded the highest area the ROC of 0.9904, showing good separability between the *family_scores* and *nonfamily_scores*.
(TIFF)

S3 Fig Receiver Operating Characteristic quantification for discriminability between *family_scores* and *nonfamily_scores* at different *t*_*e*_. For *t*_*e*_ values ranging from 0 to 2.375 (A-T), the *family_scores* (*blue*) and *nonfamily_scores* (*red*), augmented across families, are shown as normalised histograms; and the corresponding areas under the ROC are indicated. *t*_*e*_ value of 1.375 (L) yielded the highest area the ROC of 0.990, showing good separability between the *family_scores* and *nonfamily_scores*.
(TIFF)

S4 Fig Receiver Operating Characteristic quantification for discriminability between *family_scores* and *nonfamily_scores* at different *t*_*s*_. For *t*_*s*_ values ranging from 0.0 to 0.06 (A-D), the *family_scores* (*blue*) and *nonfamily_scores* (*red*), augmented across families, are shown as normalised histograms; and the corresponding areas under the ROC are indicated. *t*_*s*_ value of 0.0 (A) yielded the highest area the ROC of 0.974, showing good separability between the *family_scores* and *nonfamily_scores*.
(TIFF)

S5 Fig Performance of *ID_score* identified sites, in comparison with ungapped positions. (A) The *ID_scores* of each of the 107 families as a function of 194 alignment positions identified by *ID_score* is plotted as a heatmap in a blue-red scheme. Hotter the colour, higher is the specificity of the site to the family. The positions identified are those in which at least 10% of the families have an *ID_score* of >0.1. (B) Plotted, as a heatmap, is the *ID_scores* of the 107 families at 194 positions with the least number of gaps in the alignment. Large regions of blue, or low *ID_score*, is seen in highly conserved sites. (C) Closer snapshot of the secondary TKL group cluster as seen in Fig 5B, depicting predominantly STKR family sequences. The tree was built using 194 *ID_score* identified sites as input. (D) Closer snapshot of the secondary TKL group cluster as seen in Fig 5C, depicting predominantly STKR family sequences. The tree was built using 194 least gapped positions in the alignment as input.
(TIFF)

S6 Fig Hierarchy of STY kinase classification and active structures used to generate alignment. Sequences from 7 groups of STY kinases were organised in the hierarchy as shown. The number of families within each group, and the available crystal structures in active conformation, not more than one per family, are enlisted. For the alignment of across-group profiles, available crystal structures in active conformation, not more than one per group, were used as shown (See Methods for details).
(TIFF)

## References

[1] Krupa A, Srinivasan N. The repertoire of protein kinases encoded in the draft version of the human genome: atypical variations and uncommon domain combinations. Genome Biol. 2002;3: RESEARCH0066. Available: http://www.ncbi.nlm.nih.gov/pubmed/12537555 10.1186/gb-2002-3-12-research0066 PMC151168 12537555

[2] Hanks S. Genomic analysis of the eukaryotic protein kinase superfamily: a perspective. Genome Biol. BioMed Central; 2003;4: 111 doi: 10.1186/gb-2003-4-5-111 12734000 10.1186/gb-2003-4-5-111 PMC156577

[3] Manning G, Plowman GD, Hunter T, Sudarsanam S. Evolution of protein kinase signaling from yeast to man. Trends Biochem Sci. 2002;27: 514–20. doi: 10.1016/S0968-0004(02)02179-5 12368087 10.1016/s0968-0004(02)02179-5

[4] Taylor SS, Knighton DR, Zheng J, Ten Eyck LF, Sowadski JM. Structural Framework for the Protein Kinase Family. Annu Rev Cell Biol. Annual Reviews 4139 El Camino Way, P.O. Box 10139, Palo Alto, CA 94303–0139, USA; 1992;8: 429–462. doi: 10.1146/annurev.cb.08.110192.002241 1335745 10.1146/annurev.cb.08.110192.002241

[5] Scheid MP, Woodgett JR. Unravelling the activation mechanisms of protein kinase B/Akt. FEBS Lett. 2003;546: 108–112. doi: 10.1016/S0014-5793(03)00562-3 12829245 10.1016/s0014-5793(03)00562-3

[6] Bayliss R, Sardon T, Vernos I, Conti E. Structural basis of Aurora-A activation by TPX2 at the mitotic spindle. Mol Cell. 2003;12: 851–62. Available: http://www.ncbi.nlm.nih.gov/pubmed/14580337 14580337 10.1016/s1097-2765(03)00392-7

[7] Zhang X, Gureasko J, Shen K, Cole PA, Kuriyan J. An allosteric mechanism for activation of the kinase domain of epidermal growth factor receptor. Cell. 2006;125: 1137–49. doi: 10.1016/j.cell.2006.05.013 16777603 10.1016/j.cell.2006.05.013

[8] Brooks AJ, Dai W, O’Mara ML, Abankwa D, Chhabra Y, Pelekanos RA, et al Mechanism of Activation of Protein Kinase JAK2 by the Growth Hormone Receptor. Science (80-). 2014;344. 10.1126/science.1249783 24833397

[9] Nolen B, Taylor S, Ghosh G. Regulation of protein kinases; controlling activity through activation segment conformation. Mol Cell. 2004;15: 661–75. doi: 10.1016/j.molcel.2004.08.024 15350212 10.1016/j.molcel.2004.08.024

[10] Barford D. The mechanism of protein kinase regulation by protein phosphatases. Biochem Soc Trans. 2001;29: 385–91. Available: http://www.ncbi.nlm.nih.gov/pubmed/11497994 11497994 10.1042/bst0290385

[11] Chen RH, Sarnecki C, Blenis J. Nuclear localization and regulation of erk- and rsk-encoded protein kinases. Mol Cell Biol. American Society for Microbiology; 1992;12: 915–927. doi: 10.1128/MCB.12.3.915 1545823 10.1128/mcb.12.3.915-927.1992 PMC369523

[12] Baldin V, Ducommun B. Subcellular localisation of human wee1 kinase is regulated during the cell cycle. J Cell Sci. 1995; 2425–32. Available: http://www.ncbi.nlm.nih.gov/pubmed/7673359 7673359 10.1242/jcs.108.6.2425

[13] Griffioen G, Thevelein J. Molecular mechanisms controlling the localisation of protein kinase A. Curr Genet. Springer-Verlag; 2002;41: 199–207. doi: 10.1007/s00294-002-0308-9 12172960 10.1007/s00294-002-0308-9

[14] Trinh TB, Xiao Q, Pei D. Profiling the Substrate Specificity of Protein Kinases by On-Bead Screening of Peptide Libraries. Biochemistry. American Chemical Society; 2013;52: 5645–5655. doi: 10.1021/bi4008947 23848432 10.1021/bi4008947 PMC3773219

[15] Hemmer W, McGlone M, Tsigelny I, Taylor SS. Role of the Glycine Triad in the ATP-binding Site of cAMP-dependent Protein Kinase. J Biol Chem. American Society for Biochemistry and Molecular Biology; 1997;272: 16946–16954. doi: 10.1074/jbc.272.27.16946 9202006 10.1074/jbc.272.27.16946

[16] Zheng J, Trafny EA, Knighton DR, Xuong NH, Taylor SS, Ten Eyck LF, et al 2.2 A refined crystal structure of the catalytic subunit of cAMP-dependent protein kinase complexed with MnATP and a peptide inhibitor. Acta Crystallogr D Biol Crystallogr. 1993;49: 362–5. doi: 10.1107/S0907444993000423 15299527 10.1107/S0907444993000423

[17] Knight JDR, Qian B, Baker D, Kothary R. Conservation, variability and the modeling of active protein kinases. PLoS One. 2007;2: e982 doi: 10.1371/journal.pone.0000982 17912359 10.1371/journal.pone.0000982 PMC1989141

[18] Yang J, Kennedy EJ, Wu J, Deal MS, Pennypacker J, Ghosh G, et al Contribution of non-catalytic core residues to activity and regulation in protein kinase A. J Biol Chem. American Society for Biochemistry and Molecular Biology; 2009;284: 6241–8. doi: 10.1074/jbc.M805862200 19122195 10.1074/jbc.M805862200 PMC2649094

[19] Bjorge JD, Jakymiw A, Fujita DJ. Selected glimpses into the activation and function of Src kinase. Oncogene. Nature Publishing Group; 2000;19: 5620–5635. doi: 10.1038/sj.onc.1203923 11114743 10.1038/sj.onc.1203923

[20] Caldwell KK, Sosa M, Buckley CTC, Clark-Lewis I, Sanghera JS, Pelech SL, et al Identification of mitogen-activated protein kinase docking sites in enzymes that metabolize phosphatidylinositols and inositol phosphates. Cell Commun Signal. BioMed Central; 2006;4: 2 doi: 10.1186/1478-811X-4-2 16445858 10.1186/1478-811X-4-2 PMC1379644

[21] Lim S, Kaldis P. Cdks, cyclins and CKIs: roles beyond cell cycle regulation. Development. 2013;140. 10.1242/dev.091744 23861057

[22] Malumbres M, Manning G, Whyte DB, Martinez R, Hunter T, Sudarsanam S, et al Cyclin-dependent kinases. Genome Biol. BioMed Central; 2014;15: 122 doi: 10.1186/gb4184 25180339 10.1186/gb4184 PMC4097832

[23] Poteete AR, Rennell D, Bouvier SE. Functional significance of conserved amino acid residues. Proteins Struct Funct Genet. Wiley Subscription Services, Inc., A Wiley Company; 1992;13: 38–40. doi: 10.1002/prot.340130104 1594576 10.1002/prot.340130104

[24] Radivojac P, Clark WT, Oron TR, Schnoes AM, Wittkop T, Sokolov A, et al A large-scale evaluation of computational protein function prediction. Nat Methods. 2013;10: 221–227. doi: 10.1038/nmeth.2340 23353650 10.1038/nmeth.2340 PMC3584181

[25] Sillitoe I, Lewis T, Orengo C. Using CATH-Gene3D to Analyze the Sequence, Structure, and Function of Proteins. Current Protocols in Bioinformatics. Hoboken, NJ, USA: John Wiley & Sons, Inc.; 2015 p. 1.28.1–1.28.21. doi: 10.1002/0471250953.bi0128s50 26087950 10.1002/0471250953.bi0128s50

[26] Das S, Sillitoe I, Lee D, Lees JG, Dawson NL, Ward J, et al CATH FunFHMMer web server: protein functional annotations using functional family assignments. Nucleic Acids Res. 2015;43: W148–W153. doi: 10.1093/nar/gkv488 25964299 10.1093/nar/gkv488 PMC4489299

[27] Lichtarge O, Bourne HR, Cohen FE. An evolutionary trace method defines binding surfaces common to protein families. J Mol Biol. 1996;257: 342–58. doi: 10.1006/jmbi.1996.0167 8609628 10.1006/jmbi.1996.0167

[28] Elcock a H. Prediction of functionally important residues based solely on the computed energetics of protein structure. J Mol Biol. 2001;312: 885–896. doi: 10.1006/jmbi.2001.5009 11575940 10.1006/jmbi.2001.5009

[29] Livingstone CD, Barton GJ. Protein sequence alignments: a strategy for the hierarchical analysis of residue conservation. Comput Appl Biosci. 1993;9: 745–756. doi: 10.1073/pnas.1405652111 8143162 10.1093/bioinformatics/9.6.745

[30] Pazos F, Rausell A, Valencia A. Phylogeny-independent detection of functional residues. Bioinformatics. 2006;22: 1440–1448. doi: 10.1093/bioinformatics/btl104 16551661 10.1093/bioinformatics/btl104

[31] Casari G, Sander C, Valencia A. A method to predict functional residues in proteins. Nat Struct Biol. 1995;2: 171–178. 7749921 10.1038/nsb0295-171

[32] Wallace IM, Higgins DG. Supervised multivariate analysis of sequence groups to identify specificity determining residues. BMC Bioinformatics. 2007;8: 135 doi: 10.1186/1471-2105-8-135 17451607 10.1186/1471-2105-8-135 PMC1878507

[33] Ye K, Anton Feenstra K, Heringa J, IJzerman AP, Marchiori E. Multi-RELIEF: a method to recognize specificity determining residues from multiple sequence alignments using a Machine-Learning approach for feature weighting. Bioinformatics. 2008;24: 18–25. doi: 10.1093/bioinformatics/btm537 18024975 10.1093/bioinformatics/btm537

[34] Georgi B, Schultz J, Schliep A. Partially-supervised protein subclass discovery with simultaneous annotation of functional residues. BMC Struct Biol. 2009;9: 68 doi: 10.1186/1472-6807-9-68 19857261 10.1186/1472-6807-9-68 PMC2777906

[35] Mirny L, Gelfand MS. Using orthologous and paralogous proteins to identify specificity-determining residues in bacterial transcription factors. J Mol Biol. 2002;321: 7–20. doi: 10.1016/S0022-2836(02)00587-9 12139929 10.1016/s0022-2836(02)00587-9

[36] Kalinina O V., Mironov AA, Gelfand MS, Rakhmaninova AB. Automated selection of positions determining functional specificity of proteins by comparative analysis of orthologous groups in protein families. Protein Sci. 2004;13: 443–456. doi: 10.1110/ps.03191704 14739328 10.1110/ps.03191704 PMC2286703

[37] Gaucher E a., Gu, Miyamoto MM, Benner S a. Predicting functional divergence in protein evolution by site-specific rate shifts. Trends Biochem Sci. 2002;27: 315–321. doi: 10.1016/S0968-0004(02)02094-7 12069792 10.1016/s0968-0004(02)02094-7

[38] Pei J, Cai W, Kinch LN, Grishin N V. Prediction of functional specificity determinants from protein sequences using log-likelihood ratios. Bioinformatics. 2006;22: 164–171. doi: 10.1093/bioinformatics/bti766 16278237 10.1093/bioinformatics/bti766

[39] Hannenhalli SS, Russell RB. Analysis and prediction of functional sub-types from protein sequence alignments. J Mol Biol. 2000;303: 61–76. doi: 10.1006/jmbi.2000.4036 11021970 10.1006/jmbi.2000.4036

[40] del Sol Mesa A, Pazos F, Valencia A. Automatic methods for predicting functionally important residues. J Mol Biol. 2003;326: 1289–1302. doi: 10.1016/S0022-2836(02)01451-1 12589769 10.1016/s0022-2836(02)01451-1

[41] Yu G-X, Park B-H, Chandramohan P, Munavalli R, Geist A, Samatova NF. In silico Discovery of Enzyme–Substrate Specificity-determining Residue Clusters. J Mol Biol. 2005;352: 1105–1117. doi: 10.1016/j.jmb.2005.08.008 16140329 10.1016/j.jmb.2005.08.008

[42] Chakrabarti S, Panchenko AR. Coevolution in defining the functional specificity. Proteins Struct Funct Bioinforma. 2009;75: 231–240. doi: 10.1002/prot.22239 18831050 10.1002/prot.22239 PMC2649964

[43] Mirny L, Shakhnovich EI. Universally conserved positions in protein folds: reading evolutionary signals about stability, folding kinetics and function. J Mol Biol. 1999;291: 177–96. doi: 10.1006/jmbi.1999.2911 10438614 10.1006/jmbi.1999.2911

[44] Mihalek I, Res I, Lichtarge O. A family of evolution-entropy hybrid methods for ranking protein residues by importance. J Mol Biol. 2004;336: 1265–82. doi: 10.1016/j.jmb.2003.12.078 15037084 10.1016/j.jmb.2003.12.078

[45] Ye K, Lameijer E-WM, Beukers MW, IJzerman AP. A two-entropies analysis to identify functional positions in the transmembrane region of class A G protein-coupled receptors. Proteins Struct Funct Bioinforma. 2006;63: 1018–1030. doi: 10.1002/prot.20899 16532452 10.1002/prot.20899

[46] Pirovano W, Feenstra KA, Heringa J. Sequence comparison by sequence harmony identifies subtype-specific functional sites. Nucleic Acids Res. Oxford University Press; 2006;34: 6540–8. doi: 10.1093/nar/gkl901 17130172 10.1093/nar/gkl901 PMC1702503

[47] Mayer KM, McCorkle SR, Shanklin J. Linking enzyme sequence to function using Conserved Property Difference Locator to identify and annotate positions likely to control specific functionality. BMC Bioinformatics. 2005;6: 284 doi: 10.1186/1471-2105-6-284 16318626 10.1186/1471-2105-6-284 PMC1326233

[48] Donald JE, Shakhnovich EI. Determining functional specificity from protein sequences. Bioinformatics. Oxford University Press; 2005;21: 2629–2635. doi: 10.1093/bioinformatics/bti396 15797914 10.1093/bioinformatics/bti396

[49] Li L, Shakhnovich EI, Mirny L. Amino acids determining enzyme-substrate specificity in prokaryotic and eukaryotic protein kinases. Proc Natl Acad Sci U S A. 2003;100: 4463–4468. doi: 10.1073/pnas.0737647100 12679523 10.1073/pnas.0737647100 PMC153578

[50] Sankararaman S, Sjölander K. INTREPID—INformation-theoretic TREe traversal for Protein functional site IDentification. Bioinformatics. Oxford University Press; 2008;24: 2445–52. doi: 10.1093/bioinformatics/btn474 18776193 10.1093/bioinformatics/btn474 PMC2572704

[51] Kannan N, Neuwald AF. Evolutionary constraints associated with functional specificity of the CMGC protein kinases MAPK, CDK, GSK, SRPK, DYRK, and CK2alpha. Protein Sci. Wiley-Blackwell; 2004;13: 2059–2077. doi: 10.1110/ps.04637904 15273306 10.1110/ps.04637904 PMC2279817

[52] Neuwald AF, Kannan N, Poleksic A, Hata N, Liu JS. Ran’s C-terminal, basic patch, and nucleotide exchange mechanisms in light of a canonical structure for Rab, Rho, Ras, and Ran GTPases. Genome Res. 2003;13: 673–692. doi: 10.1101/gr.862303 12671004 10.1101/gr.862303 PMC430177

[53] de Juan D, Pazos F, Valencia A. Emerging methods in protein co-evolution. Nat Rev Genet. 2013;14: 249–61. doi: 10.1038/nrg3414 23458856 10.1038/nrg3414

[54] Donald JE, Shakhnovich EI. SDR: a database of predicted specificity-determining residues in proteins. Nucleic Acids Res. 2009;37: D191–D194. doi: 10.1093/nar/gkn716 18927118 10.1093/nar/gkn716 PMC2686543

[55] Chakrabarti S, Bryant SH, Panchenko AR. Functional Specificity Lies within the Properties and Evolutionary Changes of Amino Acids. J Mol Biol. 2007;373: 801–810. doi: 10.1016/j.jmb.2007.08.036 17868687 10.1016/j.jmb.2007.08.036 PMC2605514

[56] Kalinina O V, Gelfand MS, Russell RB. Combining specificity determining and conserved residues improves functional site prediction. BMC Bioinformatics. 2009;10: 174 doi: 10.1186/1471-2105-10-174 19508719 10.1186/1471-2105-10-174 PMC2709924

[57] Reva B, Antipin Y, Sander C. Determinants of protein function revealed by combinatorial entropy optimization. Genome Biol. 2007;8: R232 doi: 10.1186/gb-2007-8-11-r232 17976239 10.1186/gb-2007-8-11-r232 PMC2258190

[58] Capra JA, Singh M. Characterization and prediction of residues determining protein functional specificity. Bioinformatics. 2008;24: 1473–1480. doi: 10.1093/bioinformatics/btn214 18450811 10.1093/bioinformatics/btn214 PMC2718669

[59] Kannan N, Neuwald AF. Did protein kinase regulatory mechanisms evolve through elaboration of a simple structural component? J Mol Biol. 2005;351: 956–972. doi: 10.1016/j.jmb.2005.06.057 16051269 10.1016/j.jmb.2005.06.057

[60] Kannan N, Haste N, Taylor SS, Neuwald AF. The hallmark of AGC kinase functional divergence is its C-terminal tail, a cis-acting regulatory module. Proc Natl Acad Sci U S A. 2007;104: 1272–7. doi: 10.1073/pnas.0610251104 17227859 10.1073/pnas.0610251104 PMC1783090

[61] Hanks S, Hunter T. The eukaryotic protein kinase superfamily: kinase (catalytic) domain structure and classification. FASEB J. 1995;9: 576–596. Available: http://files/542/Hanks,Hunter—1995—Theeukaryoticproteinkinasesuperfamilykinase(catalytic)domainstructureandclassification.pdf 7768349

[62] Manning G, Whyte DB, Martinez R, Hunter T, Sudarsanam S. The protein kinase complement of the human genome. Science. 2002;298: 1912–34. doi: 10.1126/science.1075762 12471243 10.1126/science.1075762

[63] Hanks S. Eukaryotic protein kinases. Curr Opin Struct Biol. 1991;1: 369–383. doi: 10.1016/0959-440X(91)90035-R

[64] Hanks S, Hunter T. The eukaryotic protein kinase superfamily: (catalytic) domam structure and classification of the. The FASEB. 1995;9: 576–596. Available: http://files/549/Hanks,Hunter—1995—Theeukaryoticproteinkinasesuperfamily(catalytic)domamstructureandclassificationofthe.pdf 7768349

[65] Hanks S, Quinn A, Hunter T. The protein kinase family: conserved features and deduced phylogeny of the catalytic domains. Science (80-). 1988;241: 42–52. doi: 10.1126/science.3291115 3291115 10.1126/science.3291115

[66] Hunter T, Plowman GD. The protein kinases of budding yeast: six score and more. Trends Biochem Sci. 1997;22: 18–22. Available: http://www.ncbi.nlm.nih.gov/pubmed/9020587 10.1016/s0968-0004(96)10068-2 9020587

[67] Altschul SF, Gish W, Miller W, Myers EW, Lipman DJ. Basic local alignment search tool. J Mol Biol. 1990;215: 403–10. doi: 10.1016/S0022-2836(05)80360-2 2231712 10.1016/S0022-2836(05)80360-2

[68] The UniProt Consortium Consortium TU. UniProt: a hub for protein information. Nucleic Acids Res. 2014;43: D204–212. doi: 10.1093/nar/gku989 25348405 10.1093/nar/gku989 PMC4384041

[69] Finn RD, Coggill P, Eberhardt RY, Eddy SR, Mistry J, Mitchell AL, et al The Pfam protein families database: towards a more sustainable future. Nucleic Acids Res. Oxford University Press; 2016;44: D279–85. doi: 10.1093/nar/gkv1344 26673716 10.1093/nar/gkv1344 PMC4702930

[70] Sigrist CJA, De Castro E, Langendijk-Genevaux PS, Le Saux V, Bairoch A, Hulo N. ProRule: a new database containing functional and structural information on PROSITE profiles. Bioinformatics. 2005;21: 4060–6. doi: 10.1093/bioinformatics/bti614 16091411 10.1093/bioinformatics/bti614

[71] Katoh K, Misawa K, Kuma K, Miyata T. MAFFT: a novel method for rapid multiple sequence alignment based on fast Fourier transform. Nucleic Acids Res. 2002;30: 3059–66. Available: http://www.ncbi.nlm.nih.gov/pubmed/12136088 12136088 10.1093/nar/gkf436 PMC135756

[72] Li W, Godzik A. Cd-hit: a fast program for clustering and comparing large sets of protein or nucleotide sequences. Bioinformatics. 2006;22: 1658–9. doi: 10.1093/bioinformatics/btl158 16731699 10.1093/bioinformatics/btl158

[73] Taylor WR. The classification of amino acid conservation. J Theor Biol. Academic Press; 1986;119: 205–218. doi: 10.1016/S0022-5193(86)80075-3 3461222 10.1016/s0022-5193(86)80075-3

[74] Zvelebil MJ, Barton GJ, Taylor WR, Sternberg MJEE. Prediction of protein secondary structure and active sites using the alignment of homologous sequences. J Mol Biol. Academic Press; 1987;195: 957–961. doi: 10.1016/0022-2836(87)90501-8 3656439 10.1016/0022-2836(87)90501-8

[75] Shenkin PS, Erman B, Mastrandrea LD. Information-theoretical entropy as a measure of sequence variability. Proteins. 1991;11: 297–313. doi: 10.1002/prot.340110408 1758884 10.1002/prot.340110408

[76] Sander C, Schneider R. Database of homology-derived protein structures and the structural meaning of sequence alignment. Proteins. 1991;9: 56–68. doi: 10.1002/prot.340090107 2017436 10.1002/prot.340090107

[77] Price MN, Dehal PS, Arkin AP. FastTree: computing large minimum evolution trees with profiles instead of a distance matrix. Mol Biol Evol. Oxford University Press; 2009;26: 1641–1650. doi: 10.1093/molbev/msp077 19377059 10.1093/molbev/msp077 PMC2693737

[78] Finn RD, Miller BL, Clements J, Bateman A. iPfam: a database of protein family and domain interactions found in the Protein Data Bank. Nucleic Acids Res. 2014;42: D364–73. doi: 10.1093/nar/gkt1210 24297255 10.1093/nar/gkt1210 PMC3965099

[79] Sheridan DL, Kong Y, Parker SA, Dalby KN, Turk BE. Substrate discrimination among mitogen-activated protein kinases through distinct docking sequence motifs. J Biol Chem. American Society for Biochemistry and Molecular Biology; 2008;283: 19511–20. doi: 10.1074/jbc.M801074200 18482985 10.1074/jbc.M801074200 PMC2443660

[80] Tanoue T, Adachi M, Moriguchi T, Nishida E. A conserved docking motif in MAP kinases common to substrates, activators and regulators. Nat Cell Biol. 2000;2: 110–6. doi: 10.1038/35000065 10655591 10.1038/35000065

[81] Chang CI, Xu B, Akella R, Cobb MH, Goldsmith EJ. Crystal structures of MAP kinase p38 complexed to the docking sites on its nuclear substrate MEF2A and activator MKK3b. Mol Cell. 2002;9: 1241–9. Available: http://www.ncbi.nlm.nih.gov/pubmed/12086621 12086621 10.1016/s1097-2765(02)00525-7

[82] Lee T, Hoofnagle AN, Kabuyama Y, Stroud J, Min X, Goldsmith EJ, et al Docking motif interactions in MAP kinases revealed by hydrogen exchange mass spectrometry. Mol Cell. 2004;14: 43–55. Available: http://www.ncbi.nlm.nih.gov/pubmed/15068802 15068802 10.1016/s1097-2765(04)00161-3

[83] Liu S, Sun J-P, Zhou B, Zhang Z-Y. Structural basis of docking interactions between ERK2 and MAP kinase phosphatase 3. Proc Natl Acad Sci U S A. 2006;103: 5326–31. doi: 10.1073/pnas.0510506103 16567630 10.1073/pnas.0510506103 PMC1459354

[84] Tsatsanis C, Spandidos DA. The role of oncogenic kinases in human cancer (Review). Int J Mol Med. 2000;5: 583–90. Available: http://www.ncbi.nlm.nih.gov/pubmed/10812005 10812005 10.3892/ijmm.5.6.583

[85] Fleuren EDG, Zhang L, Wu J, Daly RJ. The kinome “at large” in cancer. Nat Rev Cancer. Nature Research; 2016;16: 83–98. doi: 10.1038/nrc.2015.18 26822576 10.1038/nrc.2015.18

[86] Simonetti FL, Tornador C, Nabau-Moretó N, Molina-Vila MA, Marino-Buslje C. Kin-Driver: a database of driver mutations in protein kinases. Database (Oxford). 2014;2014: bau104 doi: 10.1093/database/bau104 25414382 10.1093/database/bau104 PMC4237945

[87] Chatr-Aryamontri A, Breitkreutz B-J, Oughtred R, Boucher L, Heinicke S, Chen D, et al The BioGRID interaction database: 2015 update. Nucleic Acids Res. 2015;43: D470–8. doi: 10.1093/nar/gku1204 25428363 10.1093/nar/gku1204 PMC4383984

[88] Zhou L, Hosohata K, Gao S, Gu Z, Wang Z, Quigley CA, et al cGMP-Dependent Protein Kinase Iβ Interacts with p44/WDR77 to Regulate Androgen Receptor-Driven Gene Expression. Weisz A, editor. PLoS One. Public Library of Science; 2013;8: e63119–e63119. doi: 10.1371/journal.pone.0063119 23755100 10.1371/journal.pone.0063119 PMC3670919

[89] Robles-Flores M, Rendon-Huerta E, Gonzalez-Aguilar H, Mendoza-Hernandez G, Islas S, Mendoza V, et al p32 (gC1qBP) is a general protein kinase C (PKC)-binding protein; interaction and cellular localization of P32-PKC complexes in ray hepatocytes. J Biol Chem. 2002;277: 5247–55. doi: 10.1074/jbc.M109333200 11698413 10.1074/jbc.M109333200

[90] Kholodenko BN. Cell-signalling dynamics in time and space. Nat Rev Mol Cell Biol. Nature Publishing Group; 2006;7: 165–176. doi: 10.1038/nrm1838 16482094 10.1038/nrm1838 PMC1679905

[91] Moran MF, Koch CA, Anderson D, Ellis C, England L, Martin GS, et al Src homology region 2 domains direct protein-protein interactions in signal transduction. Proc Natl Acad Sci U S A. National Academy of Sciences; 1990;87: 8622–6. Available: http://www.ncbi.nlm.nih.gov/pubmed/2236073 2236073 10.1073/pnas.87.21.8622 PMC55009

[92] Gordley RM, Bugaj LJ, Lim WA. Modular engineering of cellular signaling proteins and networks. Curr Opin Struct Biol. 2016;39: 106–114. doi: 10.1016/j.sbi.2016.06.012 27423114 10.1016/j.sbi.2016.06.012 PMC5127285

[93] Pawson T, Linding R. Synthetic modular systems–reverse engineering of signal transduction. FEBS Lett. 2005;579: 1808–1814. doi: 10.1016/j.febslet.2005.02.013 15763556 10.1016/j.febslet.2005.02.013

[94] Lim WA. The modular logic of signaling proteins: building allosteric switches from simple binding domains. Curr Opin Struct Biol. 2002;12: 61–8. Available: http://www.ncbi.nlm.nih.gov/pubmed/11839491 11839491 10.1016/s0959-440x(02)00290-7

[95] Kalaivani R, de Brevern AG, Srinivasan N. Conservation of structural fluctuations in homologous protein kinases and its implications on functional sites. Proteins Struct Funct Bioinforma. 2016;84: 957–978. doi: 10.1002/prot.25044 27028938 10.1002/prot.25044

[96] Zhang Y, Skolnick J. TM-align: a protein structure alignment algorithm based on the TM-score. Nucleic Acids Res. 2005;33: 2302–2309. doi: 10.1093/nar/gki524 15849316 10.1093/nar/gki524 PMC1084323

[97] Eddy SR. Accelerated Profile HMM Searches. Pearson WR, editor. PLoS Comput Biol. Public Library of Science; 2011;7: e1002195–e1002195. doi: 10.1371/journal.pcbi.1002195 22039361 10.1371/journal.pcbi.1002195 PMC3197634

[98] Talevich E, Kannan N, Montoya JG, Liesenfeld O, Kim K, Kim K, et al Structural and evolutionary adaptation of rhoptry kinases and pseudokinases, a family of coccidian virulence factors. BMC Evol Biol. BioMed Central; 2013;13: 117 doi: 10.1186/1471-2148-13-117 23742205 10.1186/1471-2148-13-117 PMC3682881

[99] Neuwald AF. Rapid detection, classification and accurate alignment of up to a million or more related protein sequences. Bioinformatics. Oxford University Press; 2009;25: 1869–75. doi: 10.1093/bioinformatics/btp342 19505947 10.1093/bioinformatics/btp342 PMC2732367

[100] Paradis E, Claude J, Strimmer K. APE: Analyses of Phylogenetics and Evolution in R language. Bioinformatics. Oxford University Press; 2004;20: 289–290. doi: 10.1093/BIOINFORMATICS/BTG412 14734327 10.1093/bioinformatics/btg412

[101] Hastie T, Tibshirani R, Friedman J. The Elements of Statistical Learning: Data Mining, Inference, and Prediction [Internet]. 2nd ed. Springer Series in Statistics Springer New York; 2009 doi: 10.1007/978-0-387-84858-7

[102] The MathWorks I. MATLAB and Statistics Toolbox Release. Natick, Massachusetts, United States; 2012.

