# Supplementary material for: Recognition of sites of functional specialisation in all known eukaryotic protein kinase families
Source: PLoS Comput Biol. 2018 Feb 13;14(2):e1005975. doi: 10.1371/journal.pcbi.1005975 (PMC5826538; doi:10.1371/journal.pcbi.1005975)
Supplement: S1 Text — (DOCX) [file pcbi.1005975.s004.docx]

**Text S1 – Constraints in dataset selection**

For the purpose of the current study, three major constraints were applied which narrowed down the number of sequences in the dataset to 5488:

1. **The nature of reliable classification of the sequence into a family.** Since our method aims to identify family-specific functional sites in kinases, prior classification of the kinase sequences into families is a pre-requisite. The large and diverse superfamily of protein kinases was manually classified into hierarchical groups, families and subfamilies by Hanks and Hunter [5]. We retrieved all non-fragment protein kinase (Pfam domain PF00069 / PF007714) sequences of eukaryotic origin which were unambiguously mapped to a unique family by Hanks-Hunter-based KinBase [1]. This resulted in 34,881 kinase sequences of 164 families. This is described in the Dataset curation section of the main manuscript and also illustrated in Fig 1A-C.
2. **Removal of bias and redundancy within families.** Our method carries out all possible pairwise comparisons of families to identify specificity-determining residues, making it sensitive to the distribution of amino acid residues at a given alignment position in the two families of comparison. However, some families, like CDK, are highly represented due to their well-studied nature in comparison to others. Such families have a large number of sequences, from diverse phyla and organisms, which are identical or very closely related. To remove redundancy and enable comparison between families, we capped the sequence similarity within each family. Sequences within a family were clustered and representatives were chosen such that no two sequences in a family shared a sequence similarity of greater than 90%. By this method of uniform sampling from the sequence space, we were able to remove over-representation of a few sequences while still retaining the sequence divergence within a family [6,7]. This constraint resulted in 5553 sequences of 149 families.
3. **Removal of families with less than 5 sequences.** With fewer than 5 sequences in a family, the statistical power and variance in the amino acid distribution at a given alignment position is unreliable to compute uniqueness of the site. As a result, such families were discarded from the dataset resulting in a final dataset of 5488 kinase sequences of 107 families.

Unreliable classification of sequences, redundant sequence population and poor statistical representation of families are expected to increase noise in the input data. Thus, the three enlisted constraints are necessary treatments of the dataset for the method to identify the specificity-determining residues accurately.
