# Supplementary figures and images for: Recognition of sites of functional specialisation in all known eukaryotic protein kinase families
Source: PLoS Comput Biol. 2018 Feb 13;14(2):e1005975. doi: 10.1371/journal.pcbi.1005975 (PMC5826538; doi:10.1371/journal.pcbi.1005975)

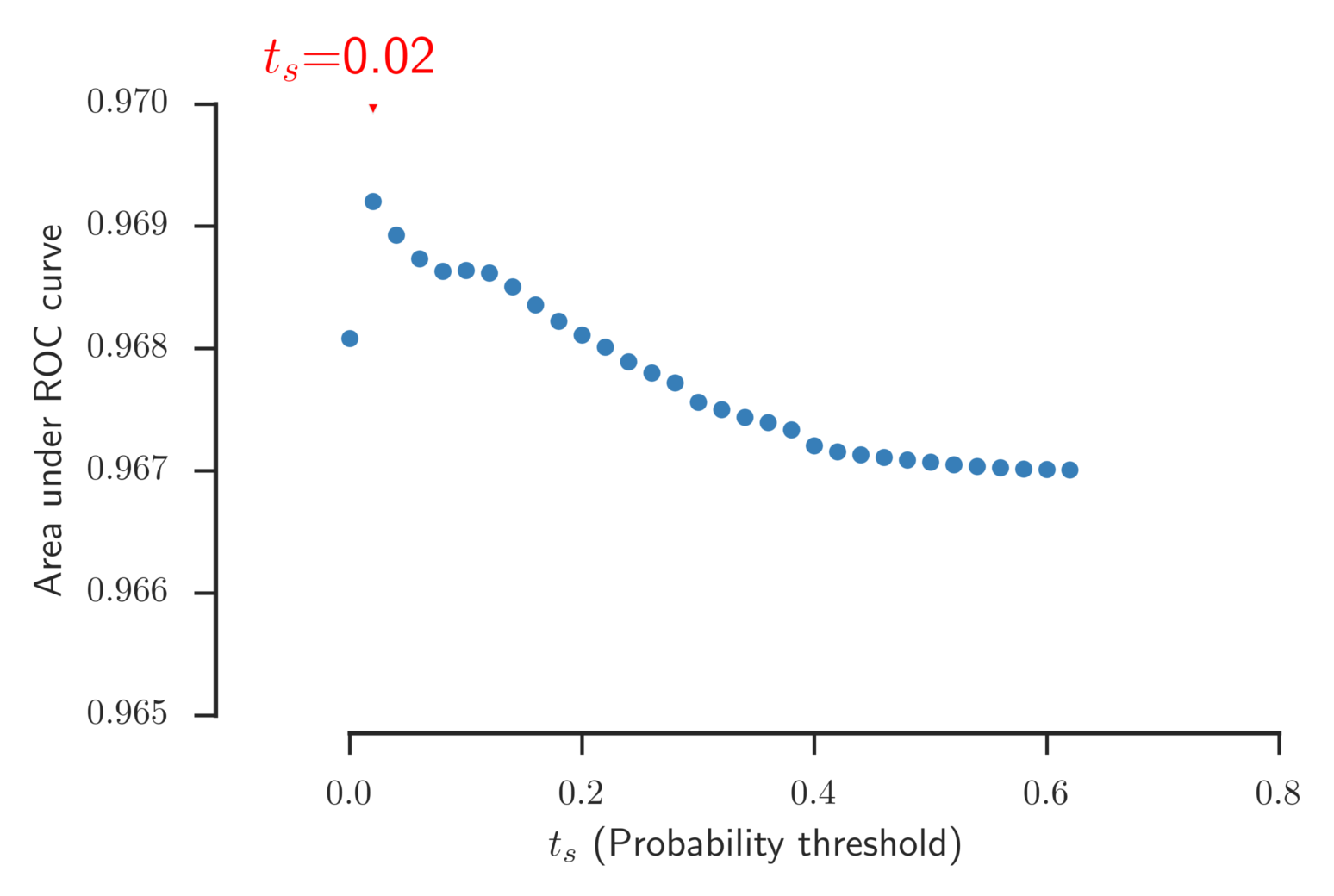

Supplement: S1 Fig — The threshold for the prob measure was optimised such that the corresponding prob_score had the highest ability to discriminate between kinase families. This was achieved by quantifying how well the family_scores were separable from the nonfamily_scores at every threshold value in terms of area under the Receiver Operating Characteristic (ROC) curve. ts, the threshold probability of obtaining the exact set of amino acids in position p in FOI when one repeatedly draws, with replacement, from the set of amino acids in the same position of nFOI was systemically tested for all possible values, and the corresponding area under the ROC curve is plotted. The maximum area under the curve (0.970) is achieved at a ts value of 0.02. This is lower than that of the ts calculated without replacement. (TIFF) [file pcbi.1005975.s005.tiff]

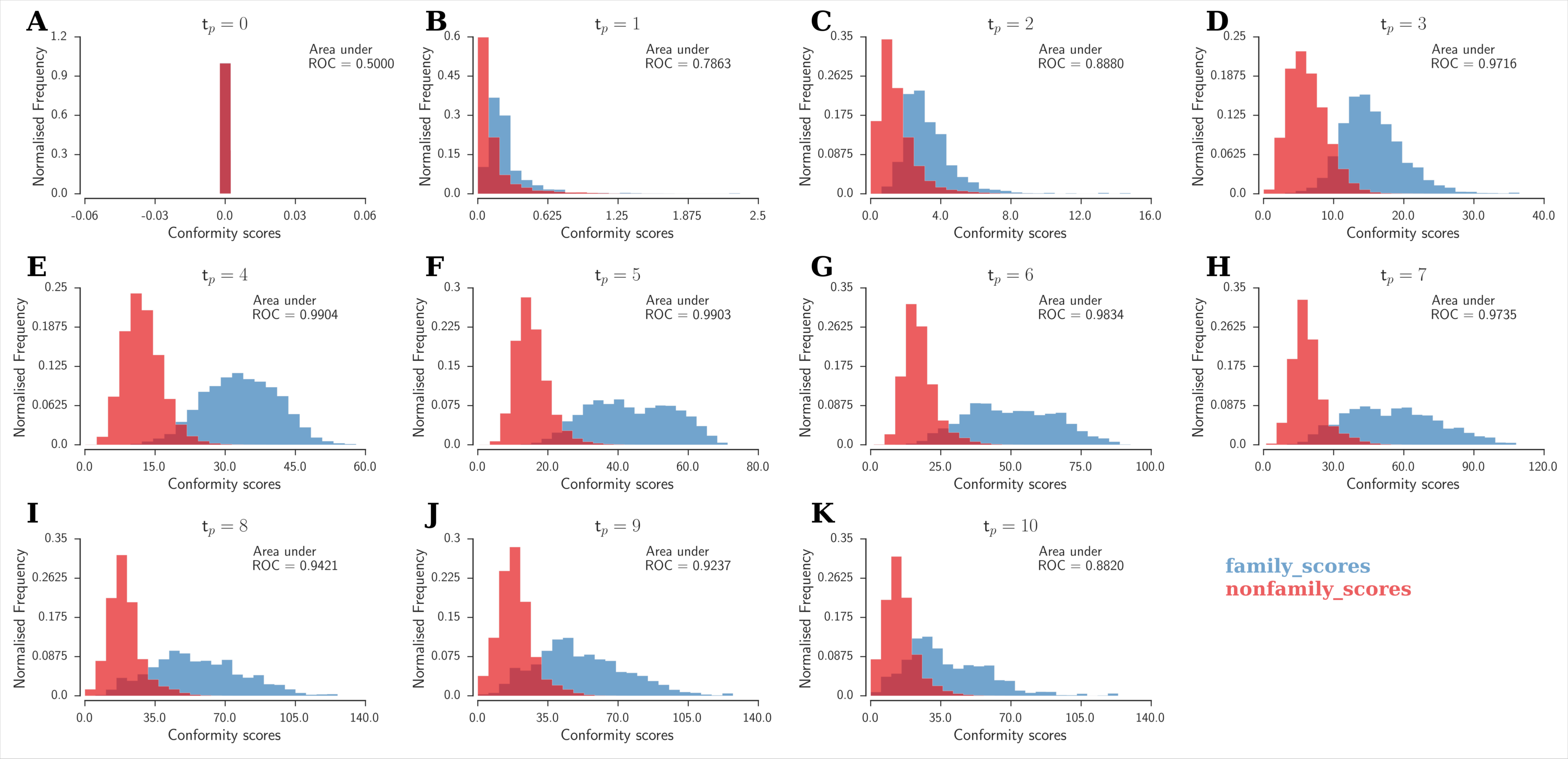

Supplement: S2 Fig — For tp values ranging from 0 to 10 (A-K), the family_scores (blue) and nonfamily_scores (red), augmented across families, are shown as normalised histograms; and the corresponding areas under the ROC are indicated. tp value of 4 (E) yielded the highest area the ROC of 0.9904, showing good separability between the family_scores and nonfamily_scores. (TIFF) [file pcbi.1005975.s006.tiff]

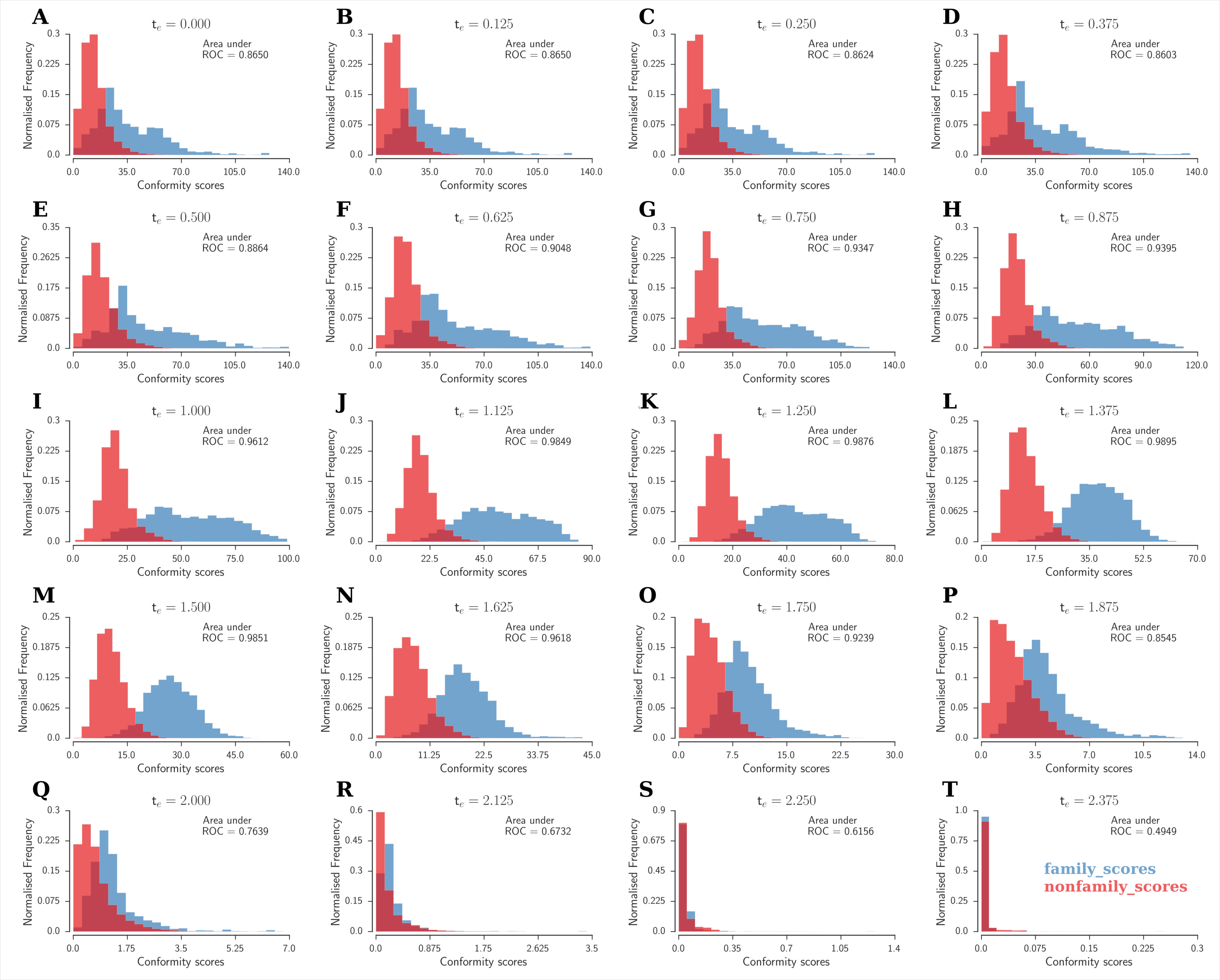

Supplement: S3 Fig — For te values ranging from 0 to 2.375 (A-T), the family_scores (blue) and nonfamily_scores (red), augmented across families, are shown as normalised histograms; and the corresponding areas under the ROC are indicated. te value of 1.375 (L) yielded the highest area the ROC of 0.990, showing good separability between the family_scores and nonfamily_scores. (TIFF) [file pcbi.1005975.s007.tiff]

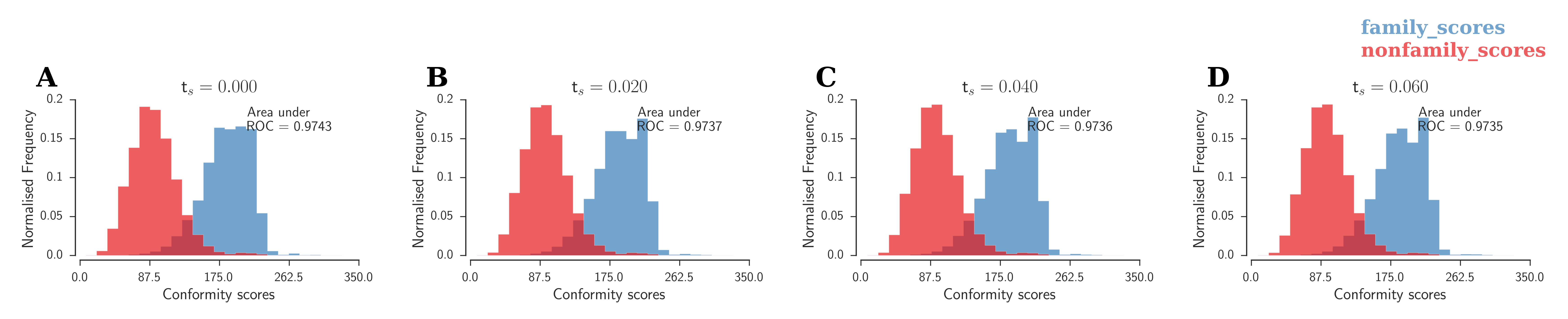

Supplement: S4 Fig — For ts values ranging from 0.0 to 0.06 (A-D), the family_scores (blue) and nonfamily_scores (red), augmented across families, are shown as normalised histograms; and the corresponding areas under the ROC are indicated. ts value of 0.0 (A) yielded the highest area the ROC of 0.974, showing good separability between the family_scores and nonfamily_scores. (TIFF) [file pcbi.1005975.s008.tiff]

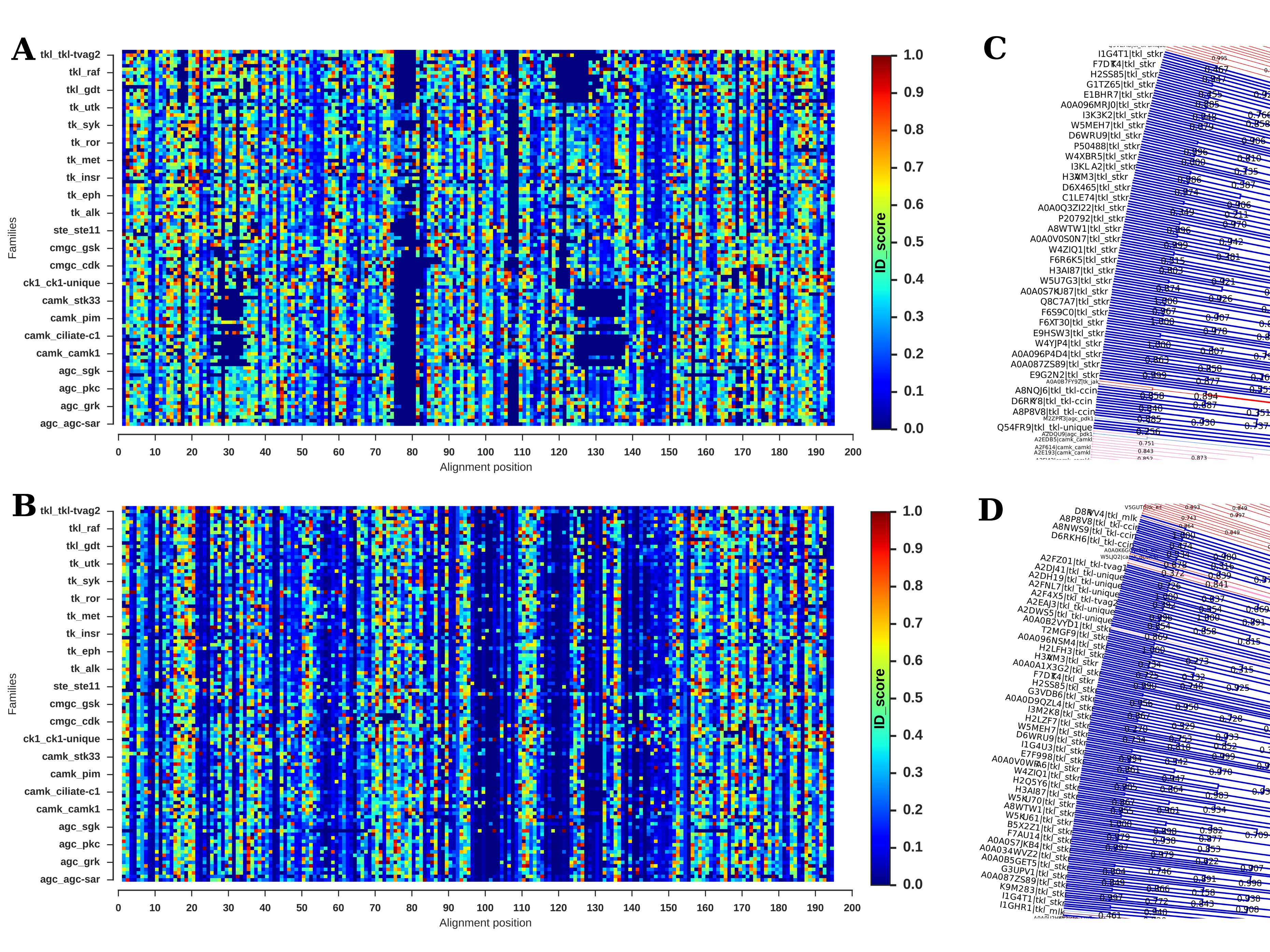

Supplement: S5 Fig — (A) The ID_scores of each of the 107 families as a function of 194 alignment positions identified by ID_score is plotted as a heatmap in a blue-red scheme. Hotter the colour, higher is the specificity of the site to the family. The positions identified are those in which at least 10% of the families have an ID_score of >0.1. (B) Plotted, as a heatmap, is the ID_scores of the 107 families at 194 positions with the least number of gaps in the alignment. Large regions of blue, or low ID_score, is seen in highly conserved sites. (C) Closer snapshot of the secondary TKL group cluster as seen in Fig 5B, depicting predominantly STKR family sequences. The tree was built using 194 ID_score identified sites as input. (D) Closer snapshot of the secondary TKL group cluster as seen in Fig 5C, depicting predominantly STKR family sequences. The tree was built using 194 least gapped positions in the alignment as input. (TIFF) [file pcbi.1005975.s009.tiff]

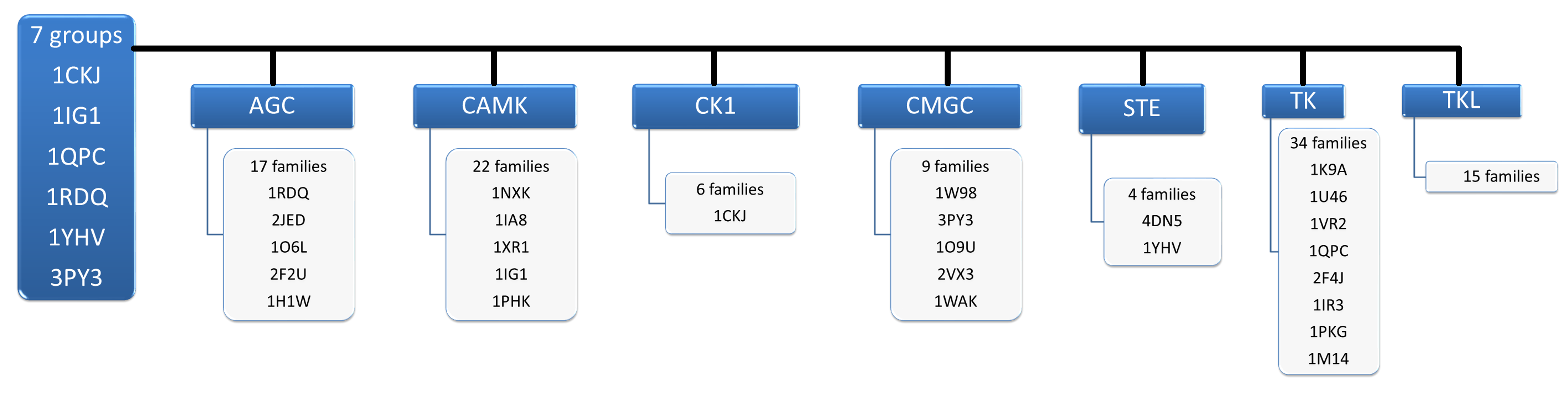

Supplement: S6 Fig — Sequences from 7 groups of STY kinases were organised in the hierarchy as shown. The number of families within each group, and the available crystal structures in active conformation, not more than one per family, are enlisted. For the alignment of across-group profiles, available crystal structures in active conformation, not more than one per group, were used as shown (See Methods for details). (TIFF) [file pcbi.1005975.s010.tiff]
